# Verapamil Alleviates Myocardial Ischemia/Reperfusion Injury by Attenuating Oxidative Stress via Activation of SIRT1

**DOI:** 10.3389/fphar.2022.822640

**Published:** 2022-02-23

**Authors:** Mi Bao, Weiyi Huang, Yang Zhao, Xinzhe Fang, Yanmei Zhang, Fenfei Gao, Danmei Huang, Bin Wang, Ganggang Shi

**Affiliations:** ^1^ Department of Pharmacology, Shantou University Medical College, Shantou, China; ^2^ Department of Clinical Pharmacy, Shantou University Medical College, Shantou, China; ^3^ Pharmaceutical Laboratory, The First Affiliated Hospital, Shantou University Medical College, Shantou, China; ^4^ Department of Cardiovascular Diseases, The First Affiliated Hospital, Shantou University Medical College, Shantou, China

**Keywords:** ischemia/reperfusion, cardioprotection, oxidative stress, SIRT1, verapamil

## Abstract

Myocardial ischemia/reperfusion (I/R) injury is a potential complication of ischemic heart disease after recanalization. One of the primary reasons for I/R injury is the excessive accumulation of reactive oxygen species (ROS) in cardiomyocytes. Verapamil, a classic calcium channel blocker, has the potential to mitigate I/R-evoked oxidative stress. However, the underlying mechanisms have not been fully elucidated. SIRT1 is an essential regulator of I/R and offers resistance to oxidative stress arising from I/R. It is still inconclusive if verapamil can reduce myocardial I/R-triggered oxidative damage through modulating SIRT1 antioxidant signaling. To verify our hypothesis, the H9c2 cardiomyocytes and the mice were treated with verapamil and then exposed to hypoxia/reoxygenation (H/R) or I/R in the presence or absence of the SIRT1 inhibitor EX527. As expected, verapamil stimulated SIRT1 antioxidant signaling evidenced by upregulation of SIRT1, FoxO1, SOD2 expressions and downregulation of Ac-FoxO1 expression *in vitro* and *in vivo*. In addition, verapamil remarkably suppressed H/R and I/R-induced oxidative stress proven by declined ROS level and MDA content. The cardioprotective actions of verapamil via SIRT1 were further confirmed in the experiments with the presence of the specific SIRT1 inhibitor EX527. We demonstrated that verapamil alleviated myocardial I/R-evoked oxidative stress partially via activation of SIRT1 antioxidant signaling. Subsequently, verapamil protected against cardiac dysfunction and myocardial infarction accompanied by oxidative stress.

## Introduction

Myocardial infarction following ischemia is responsible for the high lethality of coronary heart disease (CHD) (Kober, 2017). The current common therapies for patients with severe CHD include coronary artery bypass grafting or percutaneous coronary intervention (Lozano et al., 2020). In fact, restoring blood supply to ischemic myocardium may further aggravate tissue damage, namely myocardial ischemia/reperfusion (I/R) injury (Bi et al., 2018). Myocardial I/R injury is a complicated and multifactorial pathological process (Kawaguchi et al., 2011; Kalogeris et al., 2016; Xie et al., 2021). Of the many factors regarding the process of myocardial I/R injury, the burst of reactive oxygen species (ROS) during the reperfusion is a pivotal one with extensive well-documentation (Li et al., 2018). The excessive accumulation of ROS induces peroxidation to protein, DNA and lipid and subsequently leads to myocardial apoptosis, as observed in cardiac tissue subjected to I/R operation (Zhai et al., 2017).

While the impairment of oxygen free radicals in the hearts during I/R was recognized, increasing attention was driven in seeking antioxidants as a potential treatment option. Verapamil, a classic calcium channel blocker, plays a critical role in dilating coronary artery and is commonly used to treat angina, arrhythmias and hypertension (Klein and Weiner, 1987; Cummings et al., 1991; Tang et al., 2016). Numerous reports have also revealed that verapamil antagonizes I/R injury in different experimental models such as heart, brain, liver and so on (Korn et al., 1988; Nauta et al., 1991; Jangholi et al., 2020). Further studies have reported that mitigating oxidative stress by verapamil plays a crucial role in ameliorating myocardial I/R injury (Mandal et al., 2007; Mohan et al., 2009). Our prior study has also showed that verapamil exhibits an antioxidant action in both primary cardiomyocytes H/R and rats myocardial I/R models (Huang et al., 2009; Zhang et al., 2013). However, the underlying mechanisms have not been completely explored.

Silent information regulator of transcription 1 (SIRT1) is a deacetylase that removes the acetyl group from target proteins in the presence of nicotinamide adenine dinucleotide (NAD^+^) (Imai et al., 2000; Landry et al., 2000; Yang et al., 2013). SIRT1 is an essential regulator of myocardial I/R injury with multiple actions including resisting oxidative stress (Alcendor et al., 2007), inhibiting apoptosis (Yu et al., 2017), decreasing inflammation (Yu et al., 2016) and protecting mitochondrial function (Yang et al., 2015). Studies have identified that SIRT1 protects against hypoxia/reoxygenation (H/R) or I/R injury by attenuating oxidative stress (Becatti et al., 2012; Zhang et al., 2021). Li et al. suggested that CAPE-oNO_2_ suppressed the excessive generation of ROS via modulating the SIRT1/eNOS/NF-κB signal axis in the I/R and H/R models (Li et al., 2018). Han and colleagues also demonstrated that SIRT1 agonism inhibited pyroptosis and further alleviated myocardial I/R injury by modulating pyruvate dehydrogenase-associated glucose oxidative metabolism (Han et al., 2020). Moreover, Hsu et al. identified that in transgenic mice, modest overexpression of SIRT1 resisted myocardial I/R-evoked oxidative stress through deacetylating Ac-FoxO1 to trigger the transcription of SOD (Hsu et al., 2010). However, whether SIRT1/FoxO1 antioxidant signaling participated in verapamil’s antioxidant actions against I/R remained unclear.

Therefore, we designed the experiments to explore whether SIRT1 antioxidant signaling was associated with the amelioration of verapamil in myocardial I/R-induced oxidative damage.

## Materials and Methods

### Animals

All animal study procedures were conducted in adherence with the National Institutes of Health (NIH publication no. 86-23, revised 1996) Guidelines for the Care and Use of Laboratory Animals. The animal experimental protocols were approved by the Institutional Animal Care and Use Committee of Shantou University Medical College. Male C57BL/6J mice were purchased from Vital River Laboratory Animal Technology Co. Ltd. (Beijing, China). The mice were kept in a pathogen-free environment with free access to sterile water and food under a cycle of 12 h:12 h light-dark at 25°C.

### Reagents

Verapamil, Evans blue, triphenyltetrazolium chloride (TTC) and 2′,7′-dichlorodihydrofluorescein diacetate (DCFH-DA) were purchased from Sigma-Aldrich (St. Louis, MO, United States). Dihydroethidium (DHE) was purchased from Beyotime Institute of Biotechnology (Shanghai, China). Dulbecco’s modified eagle’s medium (DMEM) and fetal bovine serum (FBS) were purchased from Gibco Laboratory (NY, United States). Primary antibodies against SIRT1, FoxO1 and SOD2 were purchased from Cell Signaling Technology (MA, United States). The antibody against acetylated-forkhead box O1 (Ac-FoxO1) was purchased from Santa Cruz Biotechnology (Santa Cruz, CA, United States). Antibodies against β-actin, GAPDH were purchased from Beijing Zhongshan Goldenbridge Biotechnology Co., Ltd. (Beijing, China). The goat anti-rabbit and mouse secondary antibodies were purchased from Wuhan Boster Biotechnology Co., Ltd. (Wuhan, China). The SIRT1 inhibitor EX527 was bought from the Selleckchem (Houston, TX, United States). The kit for measuring malondialdehyde (MDA) was purchased from the Institute of Jiancheng Bioengineering (Nanjing, Jiangsu, China).

### Cell Culture and Establishment of Experimental Models

H9c2 cells obtained from American Type Culture Collection (ATCC, USA) were cultured in DMEM medium with 10% FBS, 100 U/ml of penicillin and 0.1 mg/ml streptomycin in a humidified cell incubator (95% air, 5% CO_2_) at 37°C. To construct cardiomyocytes H/R model, H9c2 cells were exposed to a hypoxic buffer containing the following reagents (mM): CaCl_2_ (0.9), MgCl_2_ (0.49), KCl (12), NaCl (137), Na lactate (20) and HEPES (4) at pH 6.2 in a hypoxic incubator (5% CO_2_, 94% N_2_, 1% O_2_) for 3 h and then reoxygenation was conducted by the replacement with normal air conditions and medium with 0.5% FBS for 1 h (Zhou et al., 2010). Verapamil (0.1, 1, 10 μM) was dissolved in the DMSO and administrated throughout the onset of H/R. To explore whether verapamil alleviated H/R-evoked oxidative stress by agitating SIRT1 antioxidant signaling axis, H9c2 cells were treated with and without 50 μM EX527 for 1 h before hypoxia, respectively.

### Preparation of Mouse Myocardial I/R Model and Experimental Design *in Vivo*

The mouse myocardial I/R model was conducted by ligating the left anterior descending coronary (LAD) following previously detailed description (Gao et al., 2009). In short, the mouse anesthetized with sodium pentobarbital (75 mg/kg, intraperitoneally injection) breathed with the aid of the ventilator. Then, we dissected the intercostal muscle of the mouse between the third and the fourth ribs to explore the heart. After accurately locating the LAD, we ligated it with an 8-0 silk suture as well as a small PE-10 polyethylene hose. Following 30 min of coronary occlusion, the heart blood flow was restored for 4 h or 24 h by removing the hose and untying the slipknot. The sham-operated mouse went under the parallel surgery, without the ligation silk tightened. Before the surgery, the mice were given different treatments. The mice were assigned into four groups randomly as following: Sham, I/R, I/R + Ver (1 mg/kg), I/R + Ver + EX527 (5 mg/kg/day, total 3 days before I/R). Verapamil was dissolved in saline and was administrated via intraperitoneal injection 20 min prior to ischemia. EX527 was firstly dissolved in DMSO and then diluted as the final concentration of 1 mg/ml with double distilled water according to the instruction.

### Assessment of ROS Generation in H9c2 Cells

The measurement of ROS production in H9c2 cells was carried out by flow cytometry with the DCFH-DA probe. In short, H9c2 cells were incubated in 1 μM DCFH-DA during reperfusion. Afterwards, the cells were rinsed with phosphate buffered saline (PBS) and then digested with trypsin. At last, the cells were rinsed with PBS thrice and examined with the BD Accuri™ C6 flow cytometry (BD Biosciences, CA, United States) at 525 nm emission wavelength and a 488 nm excitation wavelength.

### Determination of ROS Generation in Heart Tissues

The ROS level in myocardial frozen section was detected by DHE. Briefly, the 8 μm-thick tissue sections were incubated with 5 μM DHE at 37°C for 30 min in the dark. Afterwards, the sections were rinsed with PBS thrice. Finally, the ROS level of the tissue sections was observed under Olympus BX53 (Olympus, Tokyo, Japan) microscope. The mean fluorescent intensity (MFI) was quantified by Image-J (NIH, United States).

### Oxidative Damage Assessment

MDA, a product of peroxidation, was detected by the detection kit referring to the protocol of manufacture.

### Echocardiographic Measurement

Following 4 h of reperfusion, we measured cardiac systolic function of the mice with an ultrasound probe as mentioned previously (Wang et al., 2021). The ultrasound videos and images of mouse hearts were collected by Vevo LAZR photoacoustic imaging system (Fujifilm Visualsonics, Toronto, ON, Canada). Left ventricular ejection fraction (LVEF) and left ventricular fractional shortening (LVFS) were calculated with computerized algorithms.

### Myocardial Infarct Size

Myocardial infarction was detected by Evans blue and TTC double staining. After 24 h of reperfusion, the LAD was retied *in situ*. 0.5 ml of 1% Evans blue dye injected via the right atrium stained the non-ischemic myocardium blue. Subsequently the heart was quickly harvested, washed with saline and frozen at −30°C. As soon as the heart was frozen, it was cut transversely into 1 mm-thick slices and incubated in 1% TTC for 20 min at 37°C. The ischemic but viable myocardium was stained red in TTC dye, while only the infarction area was pale.

### Western Blot Analysis

The protein expressions were assessed by western blotting as aforementioned (Wang et al., 2015). In short, the total protein was extracted from H9c2 cells with RIPA lysis buffer and 2% protease inhibitor. The heart tissue was rinsed with PBS for the removal of blood and then homogenized with RIPA lysis buffer and 2% protease inhibitor. Then the heart homogenate was centrifuged at 13,000 rpm for 15 min twice and cell homogenate once at 4°C. Protein supernatant was boiled in SDS-PAGE sample loading buffer for 5 min. Protein concentration was determined with the PierceTM bicinchoninic acid (BCA) protein assay kit. SDS-PAGE was used to separate the proteins, which were then transferred onto nitrocellulose membranes. Following 1 h of blockage with 5% defatted milk, the membranes were incubated with antibodies against SIRT1, Ac-FoxO1, FoxO1, SOD2, β-actin and GAPDH overnight at 4°C. Following washes with TBST thrice, the membranes were probed with secondary antibodies for 90 min. Subsequently, the membranes were rinsed with TBST thrice. At last, the protein bands were visualized with SuperSignal detection kit. The gel-Pro software (Media Cybernetics, United States) was used to analyze the gray value of protein bands for quantification. Densitometry of the bands were normalized to that of housekeeping genes.

### Statistical Analysis

Data were expressed as the means ± standard error of mean (SEM). Statistical analyses of differences between groups were carried out using *t*-test or one-way analysis of variance (ANOVA) followed by least significance difference (LSD). A *p* value less than 0.05 was considered as statistically significant. The SPSS software package version 19.0 (SPSS, Chicago, IL) was used for data analysis.

## Results

### Verapamil Agitated SIRT1 Antioxidant Signaling and Suppressed the ROS Overproduction in H9c2 Cells Subjected to H/R

**FIGURE 1:**
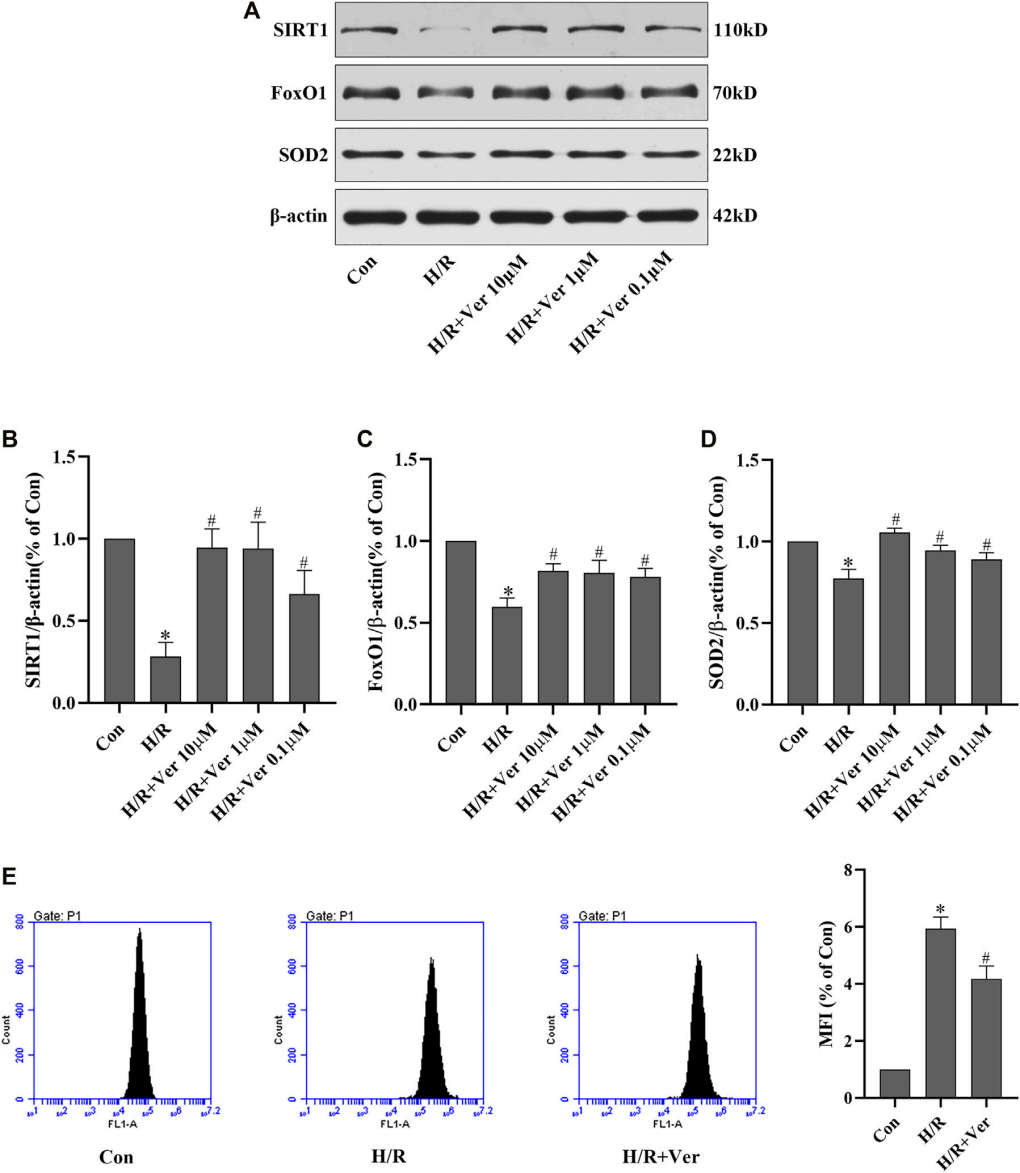
Verapamil agitated SIRT1 signaling and suppressed the ROS overproduction in H9c2 cells subjected to H/R. **(A)** Western blot to detect SIRT1, FoxO1 and SOD2 expressions. **(B–D)** Quantitative bar charts of SIRT1, FoxO1 and SOD2 expressions (*n* = 4). **(E)** DCFH-DA to detect the amount of ROS with the flow cytometry (*n* = 3). Data were expressed as means ± SEM. *p < 0.05 vs Con, ^#^ *p* < 0.05 vs H/R. Con: control, Ver: Verapamil.

Results for immunoblotting analysis manifested that the protein expressions of SIRT1, the total FoxO1 and SOD2 dropped sharply in the H/R group compared with the control group. On the contrary, verapamil treatment increased SIRT1, the total FoxO1 and SOD2 expressions in a dose-dependent fashion in the H/R conditions. (Figures 1A–D). The efficacy was the most obvious in the group treated with 10 μM verapamil, which was chosen for the following experiments. Studies have revealed that enhanced ROS production and oxidative stress are found in myocardium during I/R (Cuzzocrea et al., 2001; Jin et al., 2017). Therefore, to further investigate the antioxidant actions of verapamil in H9c2 cells H/R model, the intracellular ROS level was examined by flow cytometry with DCFH-DA staining. The H/R-triggered enhancement of ROS level was reversed by verapamil administration (Figure 1E). These results suggested that verapamil could stimulate SIRT1, FoxO1 and SOD2 and attenuate oxidative stress in H9c2 cells exposed to H/R.

### EX527 Abrogated Verapamil-Induced Stimulation of SIRT1 Antioxidant Signaling and Antioxidant Effects in H9c2 Cells Subjected to H/R

**FIGURE 2:**
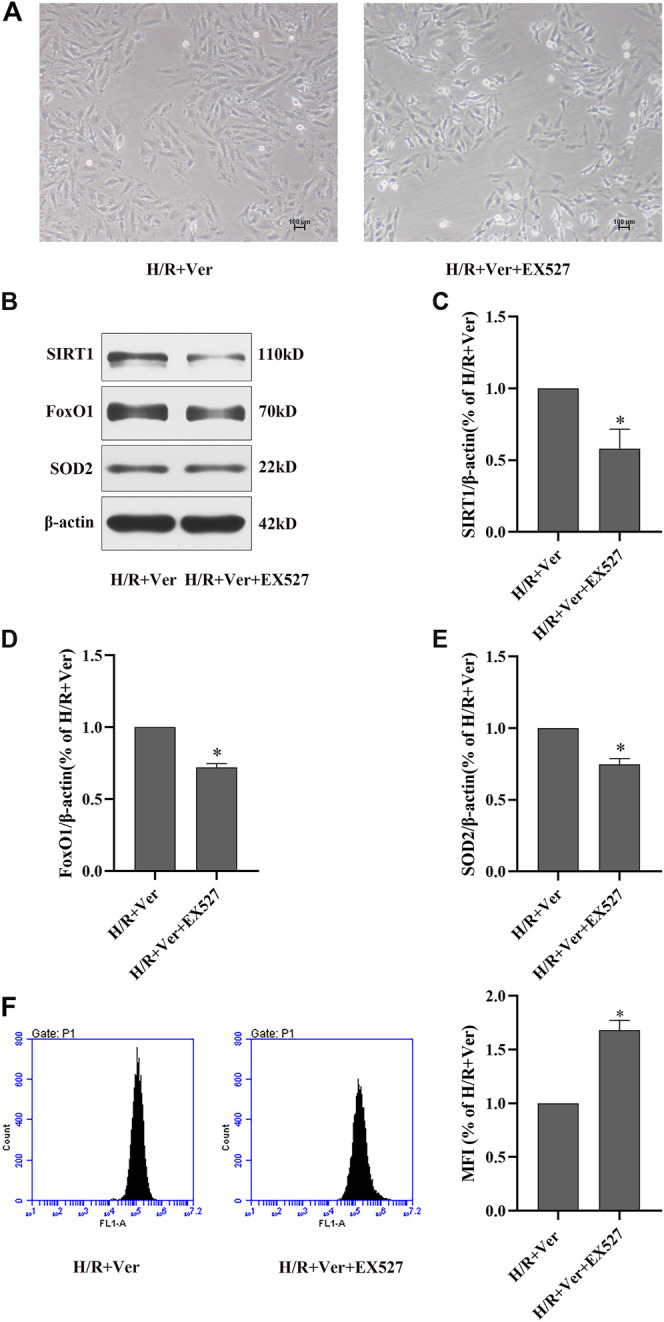
EX527 inhibited verapamil-induced SIRT1 signaling stimulation and the antioxidant effects in H9c2 cells subjected to H/R. **(A)** Representative microscopic images of H9c2 cells (100×). **(B)** Western blot to detect SIRT1, FoxO1 and SOD2 expressions. **(C–E)** Quantitative bar charts of SIRT1, FoxO1 and SOD2 expressions. **(F)** DCFH-DA to detect the amount of ROS with the flow cytometry. Data were expressed as means ± SEM (*n* = 3). *p < 0.05 vs H/R + Ver. Ver: verapamil.

To further clarify the mechanism underlying verapamil’s antioxidant effects, a selective SIRT1 inhibitor EX527 was introduced to our experiment. In comparison to verapamil-treated cells, the cells simultaneously treated with EX527 presented in shrinkage or death (Figure 2A). EX527 notably abolished the verapamil-mediated stimulation of SIRT1, the total FoxO1 and decreased the expression of SOD2 (Figures 2B–E), which validated our hypothesis that verapamil could stimulate SIRT1 antioxidant signaling in the H/R models. We hypothesized that SIRT1 antioxidant signaling was implicated in the antioxidant actions of verapamil in the H/R cardiomyocytes. Flow cytometry detected a remarkable augment in ROS formation in the cells simultaneously treated with EX527, but not in the cells treated with verapamil (Figure 2F). Taken together, verapamil dramatically reduced the H/R-induced ROS overproduction partly via activation of SIRT1 antioxidant signaling in the H9c2 cells.

### EX527 Abolished Verapamil-Induced Stimulation of SIRT1 Antioxidant Signaling and Antioxidant Effects in Mice With I/R

**FIGURE 3:**
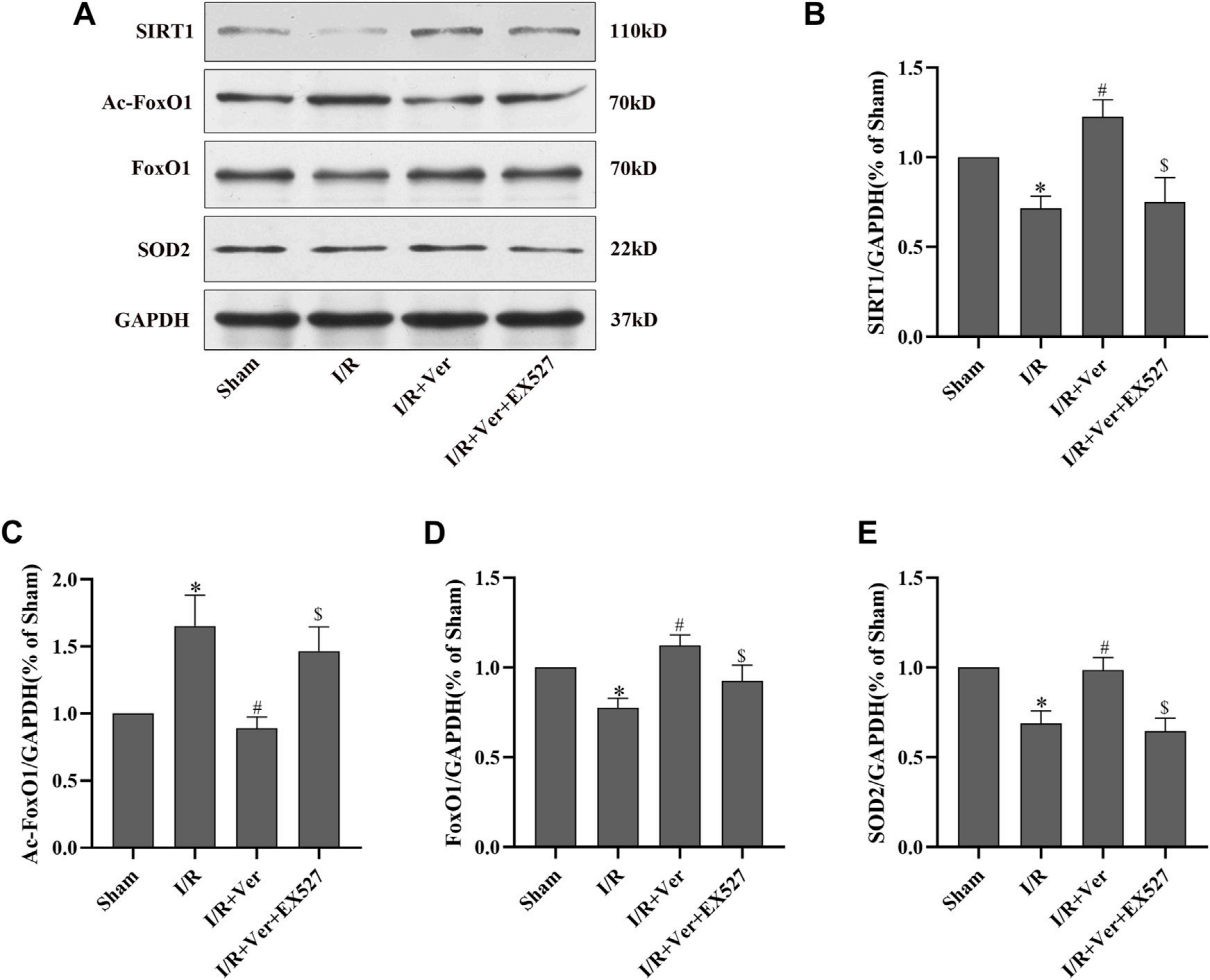
EX527 suppressed verapamil-induced SIRT1 signaling stimulation in mice with I/R. **(A)** Immunoblots showing expressions of SIRT1, Ac-FoxO1, FoxO1 and SOD2. **(B–E)** Bar graphs represented quantitation of SIRT1, Ac-FoxO1, FoxO1 and SOD2 expressions. Data were expressed as means ± SEM (*n* = 6). *p < 0.05 vs Sham, ^#^ *p* < 0.05 vs I/R, ^$^ *p* < 0.05 vs I/R + Ver. Ver: Verapamil.

Subsequently, the action of verapamil on SIRT1 antioxidant signaling was validated in the mice with I/R. The results in the mice were consistent with those obtained in the H9c2 cells. In the mice with I/R, there was observation of a downregulation of SIRT1, the total FoxO1 and SOD2 expressions but an upregulation of Ac-FoxO1 expression. Conversely, verapamil administration apparently promoted SIRT1, the total FoxO1, SOD2 expressions and suppressed Ac-FoxO1 expression (Figures 3A–E). However, the action of verapamil on SIRT1, the total FoxO1, Ac-FoxO1 and SOD2 expressions was overturned by EX527. In summary, verapamil was capable of promoting the SIRT1 antioxidant signaling in the mice with I/R.

**FIGURE 4:**
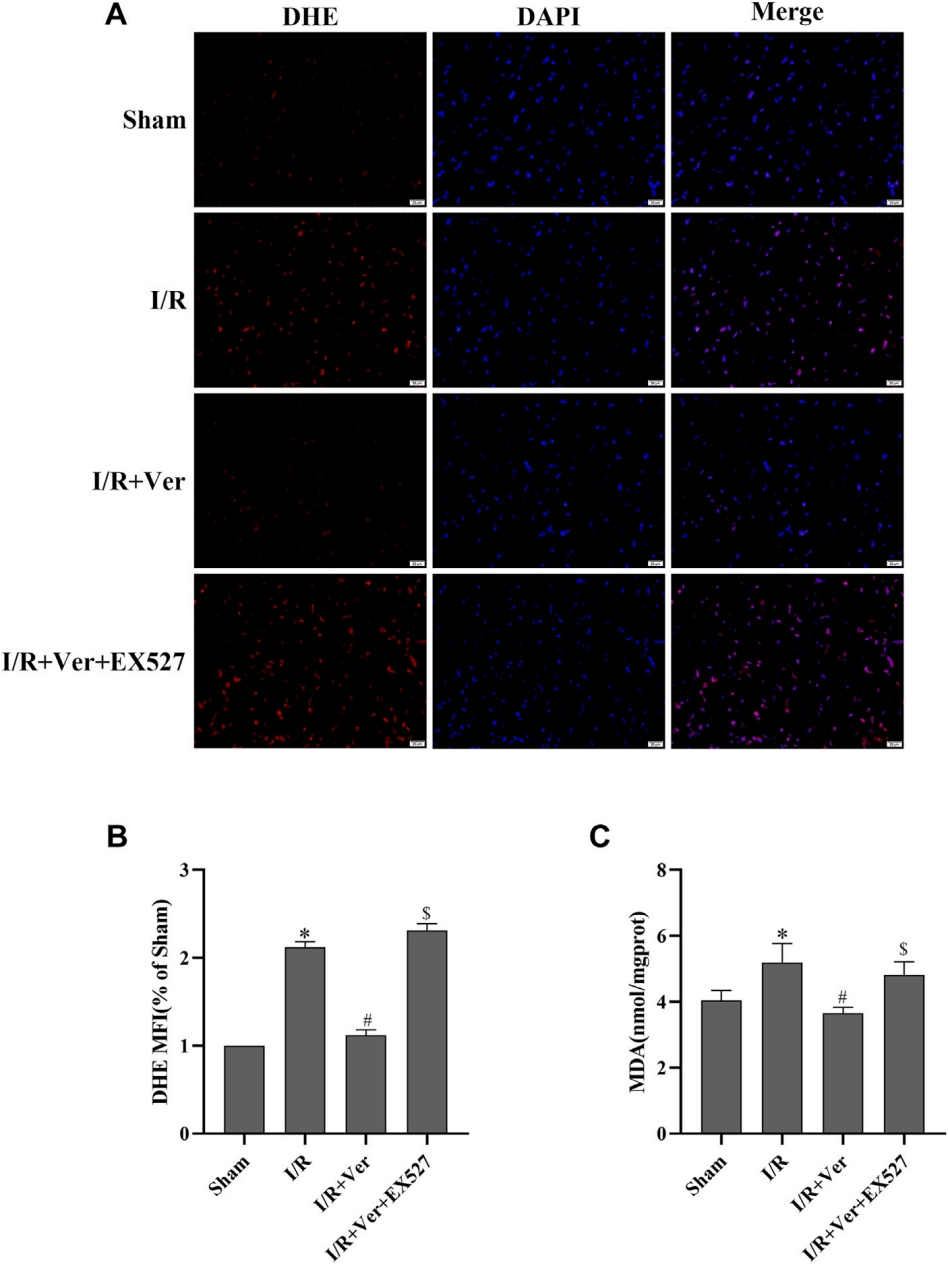
EX527 inhibited the antioxidant actions of verapamil in mice with I/R. **(A)** Representative images of DHE staining (400×) (*n* = 3). **(B)** DHE mean fluorescence intensity. **(C)** MDA content (*n* = 6). Data were expressed as means ± SEM. *p < 0.05 vs Sham, ^#^ *p* < 0.05 vs I/R, ^$^ *p* < 0.05 vs I/R + Ver. Ver: Verapamil.

The association between SIRT1 antioxidant signaling and verapamil’s antioxidant action *in vitro* reminded us that SIRT1 may also be correlated with verapamil’s antioxidant action *in vivo*. To prove this speculation, DHE was applied to the measurement of ROS production in cardiac tissue. Compared to the sham-operated mice, ROS generation was dramatically increased in the mice with I/R. In contrast, verapamil administration obviously reduced the I/R-triggered ROS production (Figures 4A,B). In addition, the MDA content was measured to estimate the level of lipid peroxidation. I/R induced an apparent upregulation of the MDA content, while verapamil administration downregulated the content of MDA (Figure 4C). In general, verapamil played an antioxidant role in myocardial I/R. SIRT1 selective inhibitor EX527 repressed the effects of verapamil in the I/R hearts, implying that SIRT1 mediated the antioxidant action of verapamil.

### EX527 Blunted the Cardioprotective Effects of Verapamil in Mice With I/R

**FIGURE 5:**
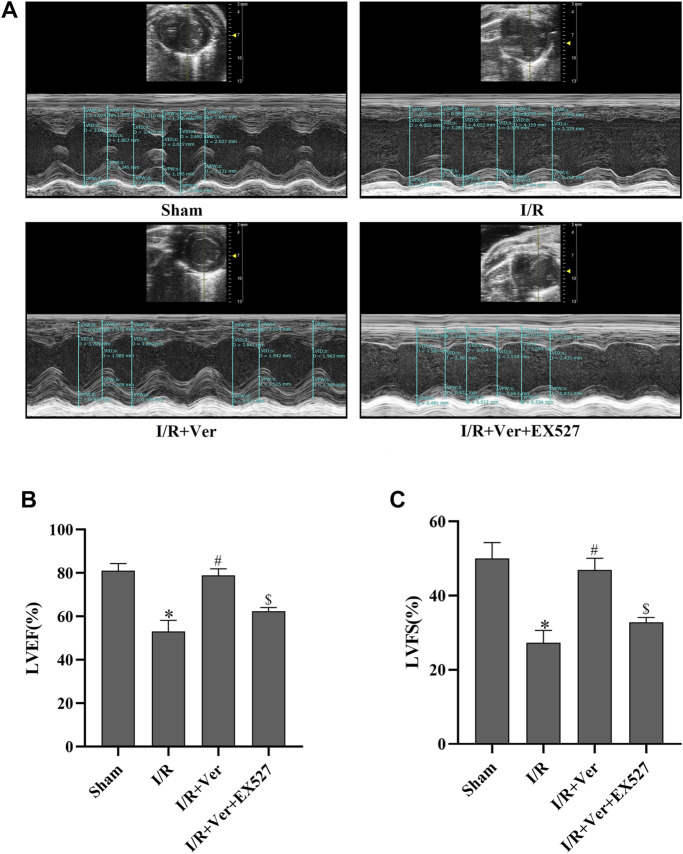
EX527 blunted the verapamil’s benefits on cardiac function in mice with I/R. **(A)** Representative M-mode echocardiogram images. **(B)** LVEF. **(C)** LVFS. Data were expressed as means ± SEM (n = 6). *p < 0.05 vs Sham, ^#^ *p* < 0.05 vs I/R, ^$^ *p* < 0.05 vs I/R + Ver. Ver: Verapamil.

**FIGURE 6:**
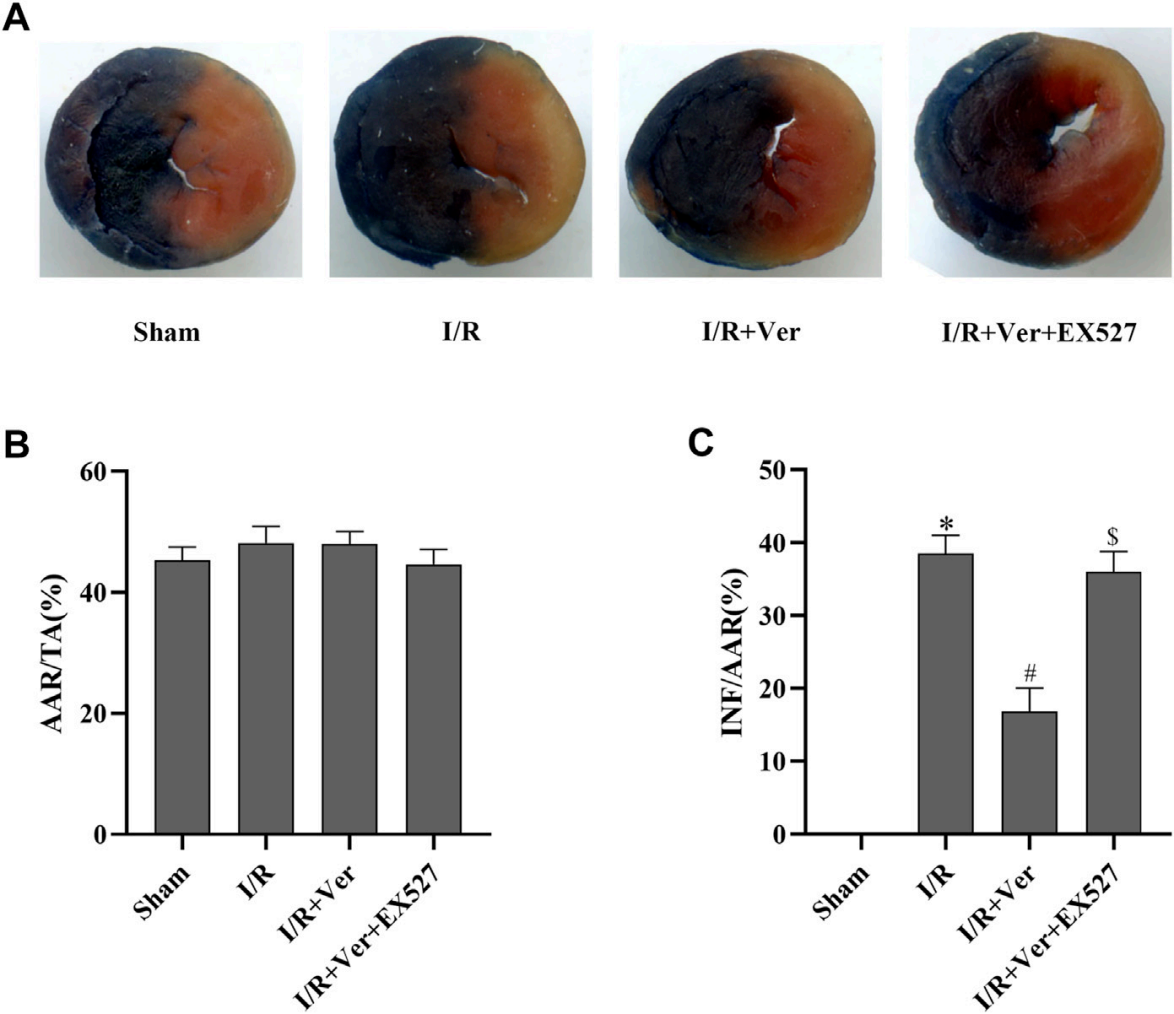
EX527 reversed verapamil’s suppression on the infarct size in mice with I/R. **(A)** Representative digital scan of cross-sectional hearts with Evans blue and TTC. **(B)** Ischemic area at risk. Bar graphs represented quantitation of ischemic area at risk (AAR) expressed as a percentage of the total area. (TA) (AAR/TA). **(C)** Myocardial infarct size. Bar graphs represented quantitation of the infarct area (INF) expressed as a percentage of AAR (INF/AAR). Data were expressed as means ± SEM (*n* = 6). *p < 0.05 vs Sham, ^#^ *p* < 0.05 vs I/R, ^$^ *p* < 0.05 vs I/R + Ver. Ver: Verapamil.

In order to ascertain that SIRT1 participated in the verapamil-mediated cardioprotective effects in the mice subjected to I/R, we assessed the cardiac function and myocardial infarct size of the mice. After 4 h of reperfusion, the mice with I/R showed a decrease in LVEF and LVFS. On the contrary, echocardiography illustrated an increase of LVEF and LVFS in verapamil treatment group compared with the I/R group (Figures 5A–C). After 24 h of reperfusion, the infarct size in the I/R group increased apparently to 38.5 ± 2.55%. In contrast, verapamil administration decreased the infarct size from 38.5 ± 2.55% to 16.8 ± 3.24% compared with the I/R group (Figures 6A–C). It was evident that verapamil was able to restore post-I/R cardiac function of the mice. However, EX527 diminished the protective effects. These results suggested that part of verapamil’s protective effects was mediated by SIRT1 antioxidant signaling pathway.

## Discussion

**FIGURE 7:**
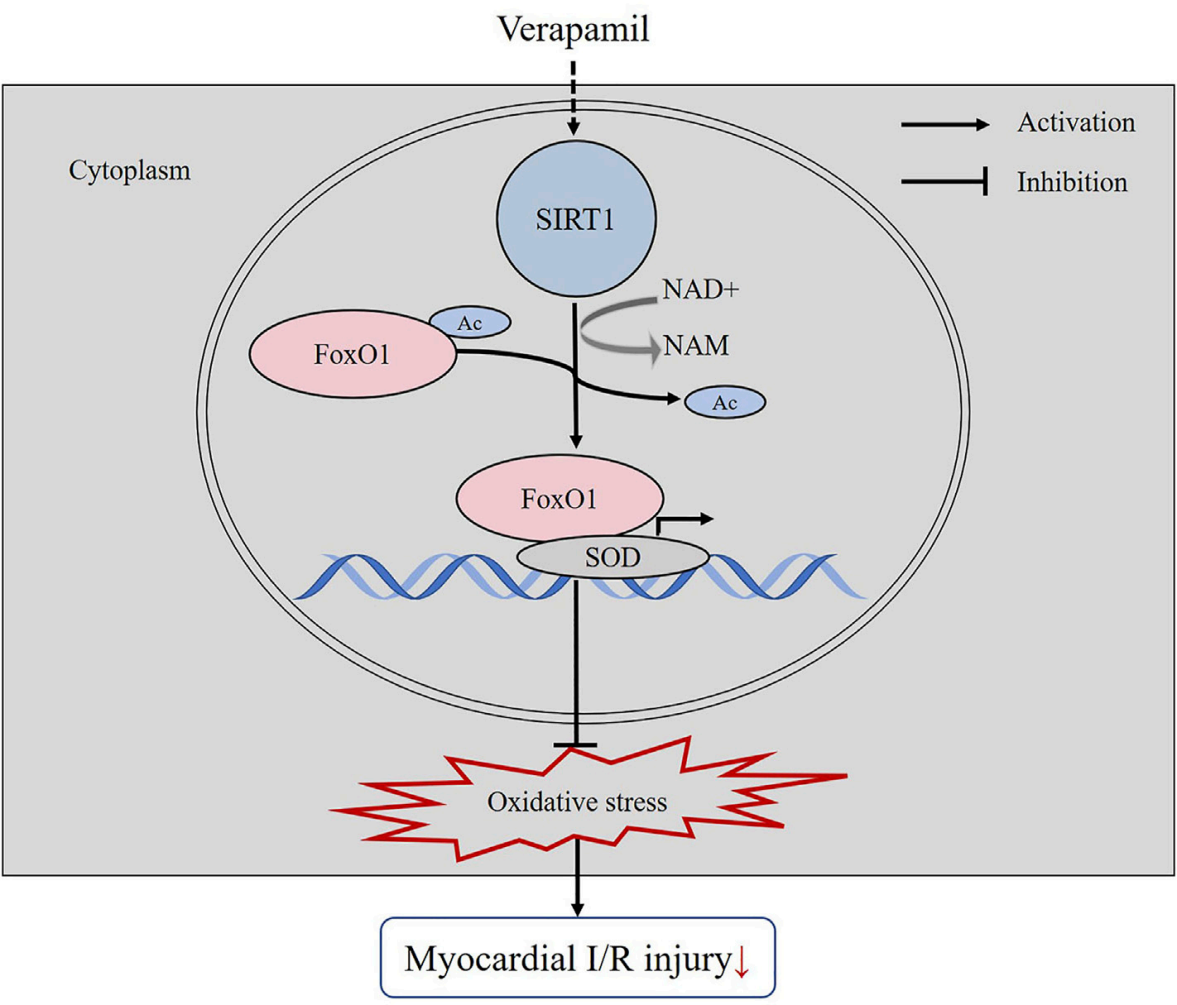
The mechanistic figure of how verapamil alleviates the oxidative stress induced by myocardial I/R.

Our experiments confirmed that verapamil had the potential to reduce oxidative stress and consequently reconstruct cardiac function in the H9c2 cells H/R and the mice I/R models. We revealed a novel molecular mechanism by which verapamil mitigated myocardial I/R-provoked oxidative stress. For the first time, our results demonstrated that verapamil’s cardioprotective action against myocardial I/R injury was partly mediated by the stimulation of SIRT1 antioxidant signaling *in vitro* and *in vivo* (Figure 7).

Oxidative stress, regarded as the primary cause of myocardial I/R injury, initiates a cascade of deleterious cellular processes including endothelial dysfunction, cardiomyocyte injury and apoptosis (Simon et al., 2021). During reperfusion, overproduction of ROS triggers cytosolic Ca^2+^ overload, which in turn disrupts the balance of Ca^2+^-dependent redox systems, resulting in a further increase of ROS (Aldakkak et al., 2011; Chang et al., 2019). Excessive ROS forces the mitochondrial permeability transition pore to open, leading to cytochrome c release from the mitochondria and eventually initiating the endogenous apoptosis pathway responsible for cell death and cardiac dysfunction (Murphy and Steenbergen, 2008). In this study, verapamil exhibited a potent antioxidant capability in the H9c2 cells H/R and the mice I/R models that verapamil treatment significantly increased the expression of antioxidant enzyme SOD2 with decreased ROS level and MDA content according to our observation. I/R-evoked massive ROS generation promoted the cardiomyocytes death and cardiac dysfunction with gross increase in the infarct size as well as decrease in LVFS and LVEF. Verapamil treatment significantly reversed this adverse phenomenon.

The sirtuin family are NAD^+^-dependent deacetylases. SIRT1, the most extensively studied sirtuin, is beneficial in cardiovascular system (Kane and Sinclair, 2018). Currently, it is well accepted that adenosine monophosphate-activated protein kinase (AMPK) is the most common pathway responsible for the activation of the majority in sirtuin family (Eid et al., 2021). AMPK enhances SIRT1 activity by increasing cellular NAD^+^ levels while SIRT1 deacetylates liver kinase B1 (LKB1), leading to phosphorylation and activation of AMPK, a positive feedback loop (Lan et al., 2008; Iwabu et al., 2010). In theory, I/R-induced Ca^2+^ overload may lead to activation of calcium/calmodulin-dependent protein kinase2 (CaMKK2) (Sung & Choi, 2012) and eventually cause the compensatory elevation of AMPK. Though the results in our study were in contradiction to the theory, presenting the downregulation of SIRT1 during I/R, Park et al. suggested a biphasic control of AMPK activity by Ca^2+^ (Park et al., 2011). Moreover, studies showed that the levels of SIRT1 and p-AMPK were significantly decreased in the infarcted myocardium of I/R-induced rats (Eid et al., 2021; Liu et al., 2021), suggesting that Ca^2+^ may indirectly influence SIRT1 signaling by regulating AMPK, of which the potential mechanisms awaited to be investigated. It has been reported that mitochondrial Ca^2+^ overload rapidly increased protein acetylation via a buildup of NADH and the inhibition of sirtuin enzymatic activity (Marcu et al., 2014). In addition, the increase of Ca^2+^ concentration in cytosol activated protein kinase Cδ (PKCδ) which negatively regulated SIRT1 expression (Lee et al., 2019). Our previous study revealed that the quaternary ammonium salt derivative of haloperidol, N-n-Butyl haloperidol iodide (F_2_), a new calcium antagonist that reduced cytosolic calcium level, inhibited the Ca^2+^-dependent translation of PKCα (Wang et al., 2010). In the present study, verapamil, a calcium channel blocker, induced the activation of SIRT1 likely via the elevation of [NAD^+^/NADH]_cyt_ ratio or the inhibition of PKC. However, the exact mechanism of SIRT1 upregulation in verapamil treatment group would be further studied in the future.

SIRT1 eliminates ROS and maintains the balance of redox system in mitochondria mainly by deacetylating Ac-FoxO1 and promoting the transcription of antioxidant genes including SOD, catalase and GSH-Px. Previously, it has been established that the deacetylation of Ac-FoxO1 via SIRT1 enabled FoxO1 to initiate the transcription of SOD, thereby counteracting ROS evoked by I/R (Hsu et al., 2010). Besides, curcumin-induced activation of SIRT1/FoxO1 signaling and diminution of mitochondrial oxidative damage showed protective effects in rat I/R hearts and H/R cardiomyocytes (Yang et al., 2013). In the current study, we discovered that verapamil potentiated SIRT1 expression, accompanied by the low expression of Ac-FoxO1 in I/R. Importantly, upregulation of SIRT1 induced by verapamil increased the expression of SOD2 which contributed to the reduction of I/R-evoked oxidative stress. Verapamil reduced oxidative stress and thereby protected the mice against myocardial I/R injury reflected by restored cardiac function and decreased infarct size. Notably, inhibiting SIRT1 by EX527 remarkably blunted the cardioprotective actions of verapamil in I/R. These results suggested that verapamil-induced stimulation of SIRT1 downregulated the acetylation of FoxO1 and exerted resistance to oxidative damage in I/R hearts.

Beyond that, we were surprised to observe that H/R and I/R could induce the downregulation of the total FoxO1 expression. Conversely, verapamil apparently enhanced the total FoxO1 expression in H/R and I/R. The results from EX527 and verapamil treated groups again supported our findings. Interestingly, verapamil downregulated Ac-FoxO1 expression but enhanced SIRT1-dependent expression of the total FoxO1 in I/R. Hsu et al. identified that the total FoxO1 was positively regulated by SIRT1 not only at the protein level but also at the mRNA level in cultured cardiomyocytes. Verapamil may augment SIRT1-dependent expression of the total FoxO1 largely by strengthening the transcription of FoxO1. Subsequently, FoxO1 could induce the expression of SOD and thereby mediate the antioxidant actions of verapamil in H/R and I/R. We verified that the stimulation of SIRT1/FoxO1 signaling pathway contributed to the beneficial effects of verapamil in myocardial I/R injury. However, the exact performance of different post-modification of FoxO1 in I/R was not elucidated. Further work was demanding to clarify the precise relationship among the total FoxO1, SIRT1 and I/R.

There are still some limitations in our study. Firstly, our *in vitro* experiments were performed in H9c2 cells which may not be able to fully mimic the function of neonatal cardiomyocytes. Secondly, the conclusion that the cardioprotective effects of verapamil in I/R were partly mediated by SIRT1 was verified using EX527. EX527 may have some inhibitory effects on SIRT2, but its selectivity is much lower than that on SIRT1. Still, the specificity of EX527 and the inhibition mechanisms of EX527 are under investigation. Despite these limitations, we believe that this study has provided important new information for the understanding of antioxidant effects of verapamil in H/R and I/R.

## Conclusion

In conclusion, our results confirm that verapamil alleviates myocardial I/R injury by inhibiting the oxidative stress in both *in vivo* and *in vitro* models. In addition, we further confirm that the cardioprotective effects of verapamil are antagonized in the presence of EX527. All of the results suggest that the effects of verapamil against I/R injury are closely related to the activation of SIRT1 antioxidant signaling. Thus, our study provides a new mechanism for verapamil in the treatment of ischemic heart disease.

## Data Availability

The original contributions presented in the study are included in the article/Supplementary Materials, further inquiries can be directed to the corresponding authors.
