# Supplementary figures and images for: Verapamil Alleviates Myocardial Ischemia/Reperfusion Injury by Attenuating Oxidative Stress via Activation of SIRT1
Source: Front Pharmacol. 2022 Feb 23;13:822640. doi: 10.3389/fphar.2022.822640 (PMC8905444; doi:10.3389/fphar.2022.822640)

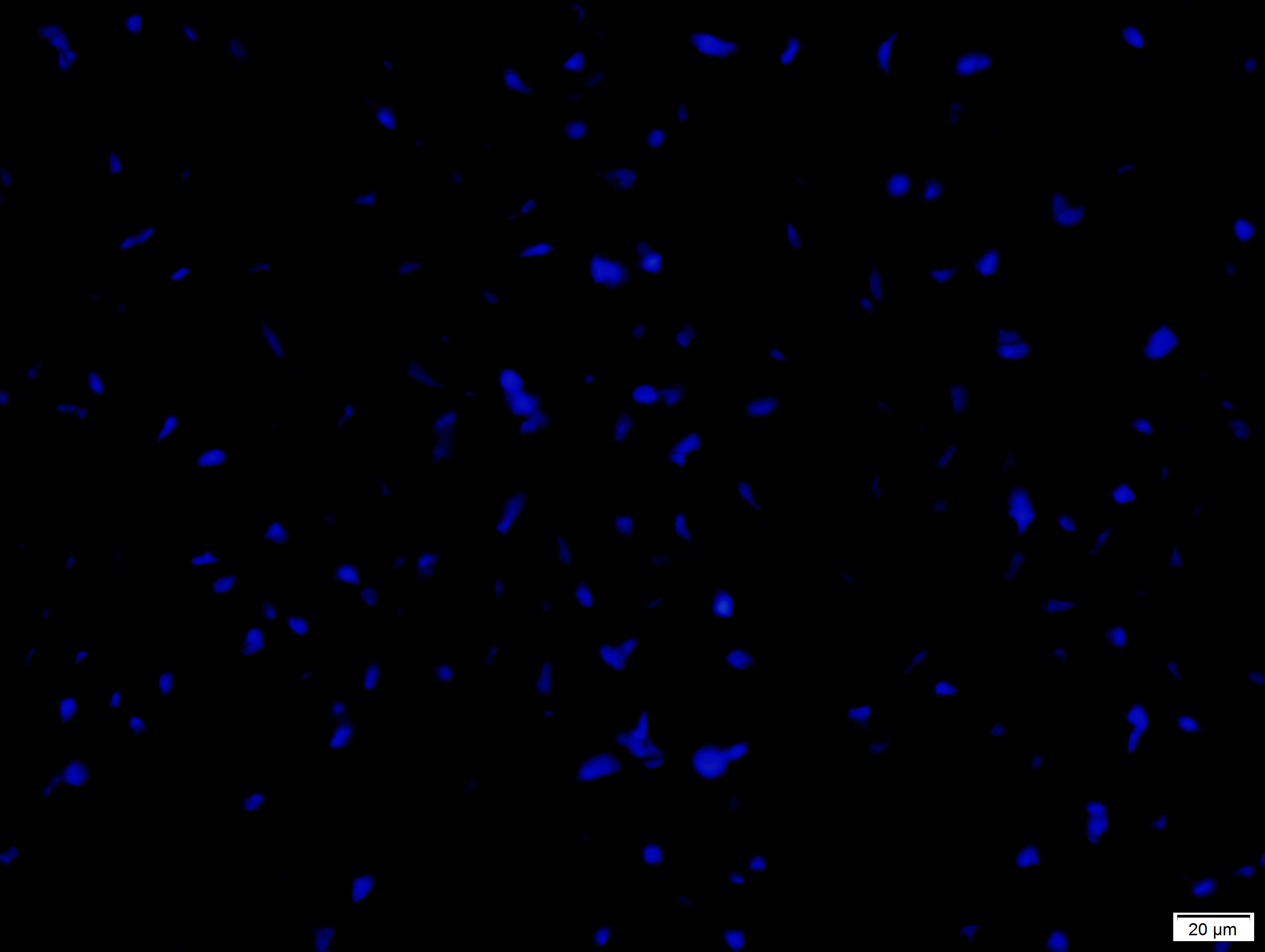

Supplement: Supplementary file 1 [file DataSheet3.ZIP › Supplement data 3/FIGURE 4/FIGURE 4A-B/microscopy images/1-400x-DAPI-2ms.tif]

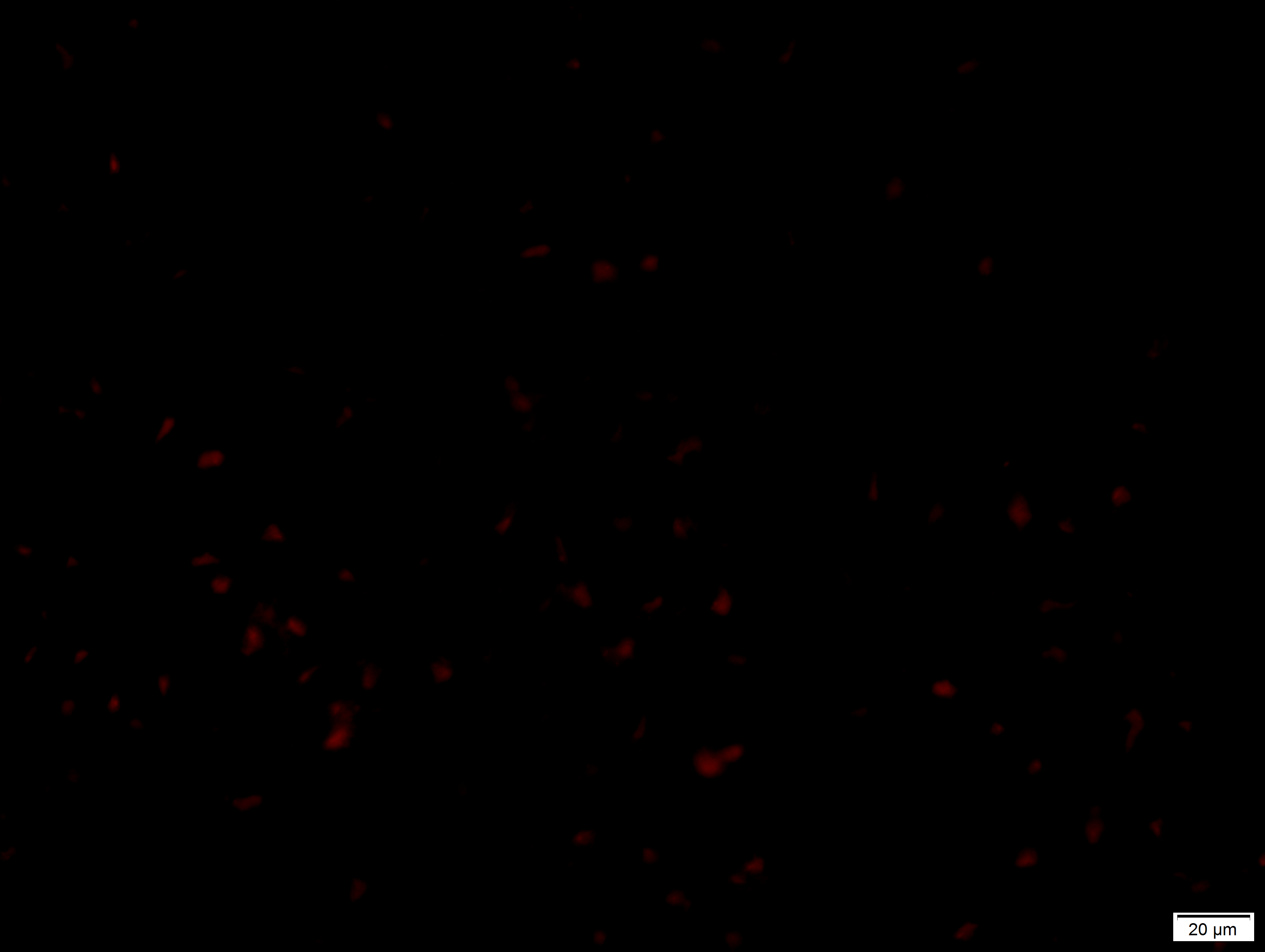

Supplement: Supplementary file 1 [file DataSheet3.ZIP › Supplement data 3/FIGURE 4/FIGURE 4A-B/microscopy images/1-400x-DHE-50ms.tif]

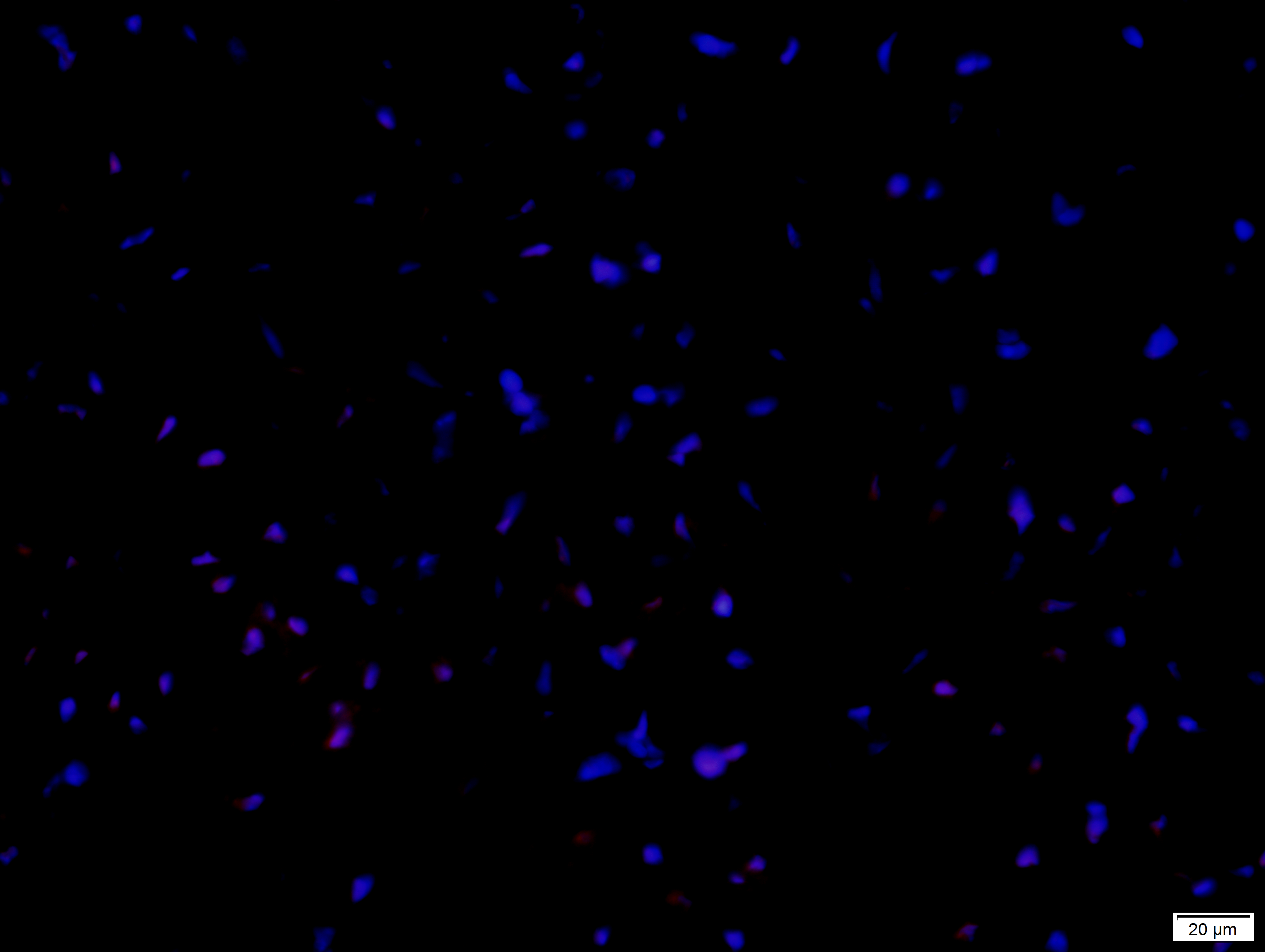

Supplement: Supplementary file 1 [file DataSheet3.ZIP › Supplement data 3/FIGURE 4/FIGURE 4A-B/microscopy images/1-400x-merge.tif]

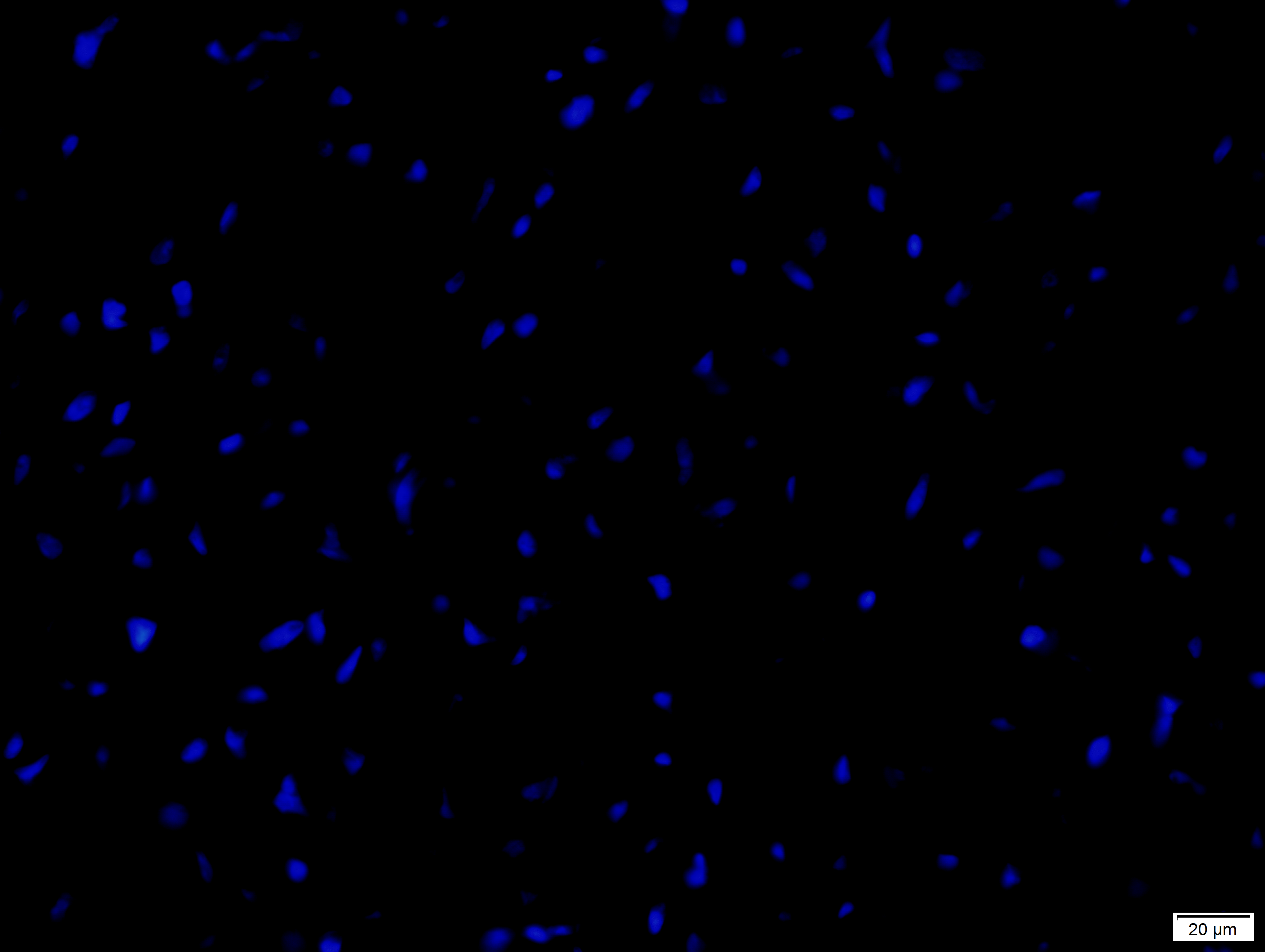

Supplement: Supplementary file 1 [file DataSheet3.ZIP › Supplement data 3/FIGURE 4/FIGURE 4A-B/microscopy images/IR-400x-DAPI-2ms.tif]

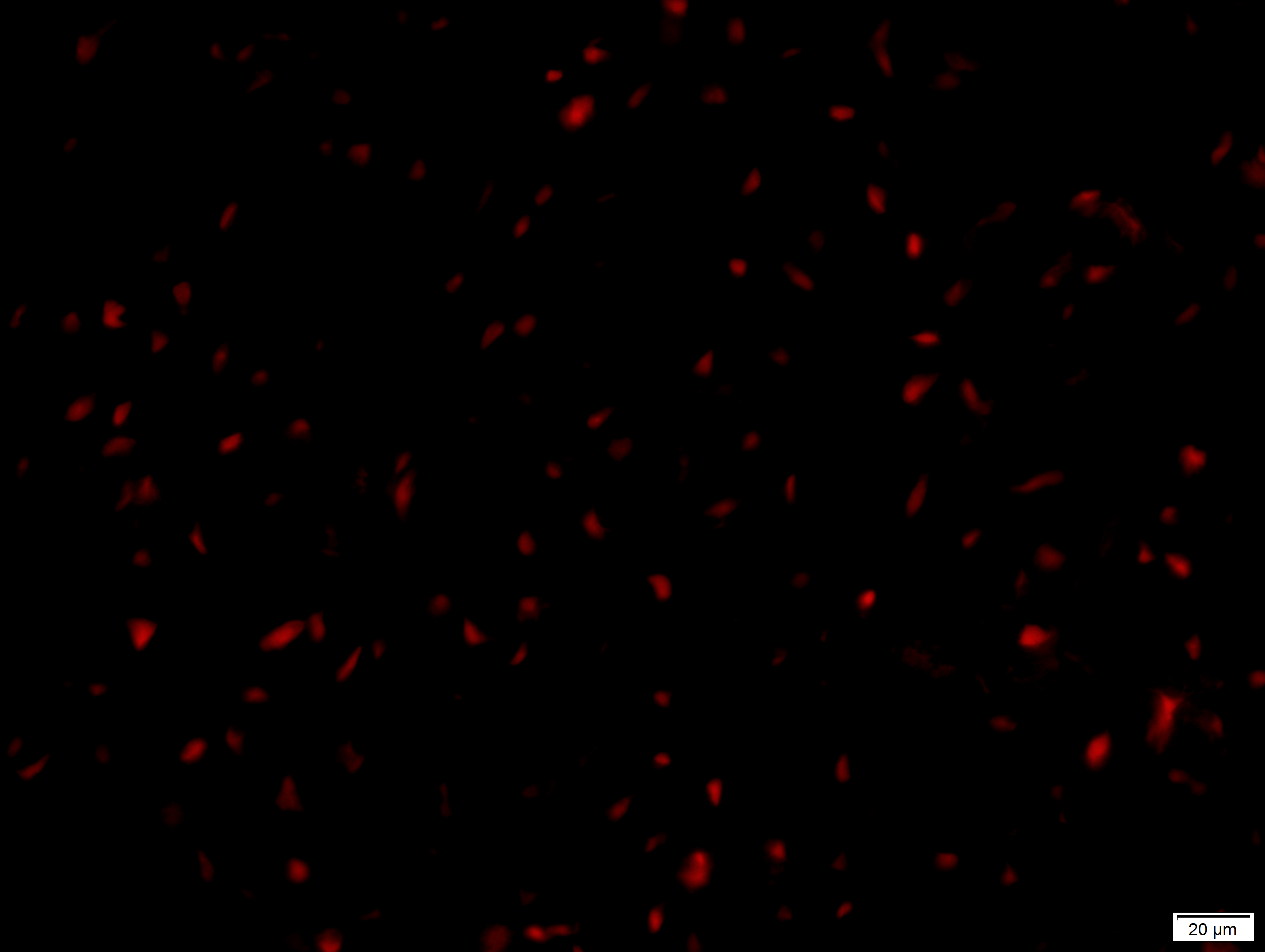

Supplement: Supplementary file 1 [file DataSheet3.ZIP › Supplement data 3/FIGURE 4/FIGURE 4A-B/microscopy images/IR-400x-DHE-50ms.tif]

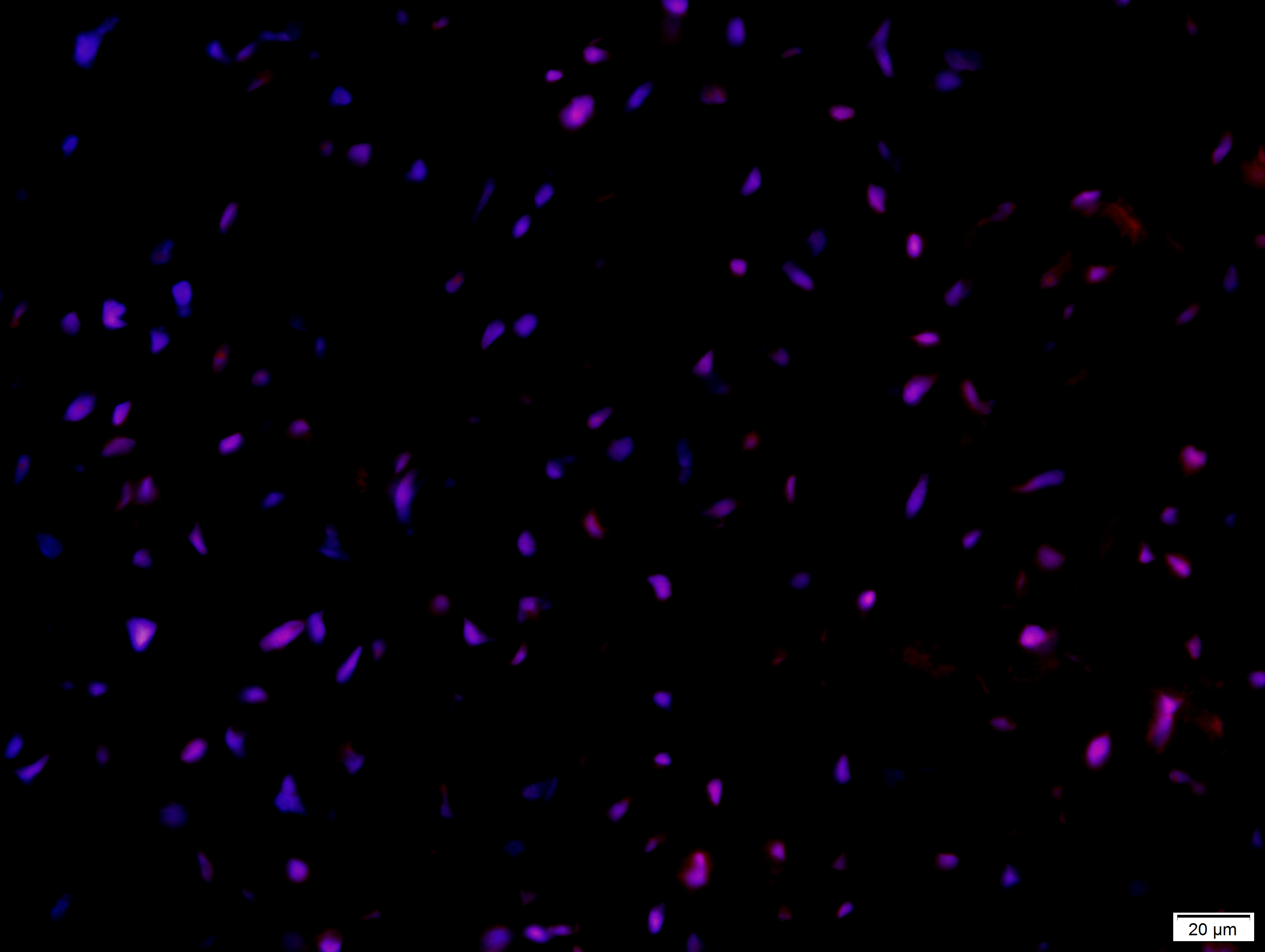

Supplement: Supplementary file 1 [file DataSheet3.ZIP › Supplement data 3/FIGURE 4/FIGURE 4A-B/microscopy images/IR-400x-merge.tif]

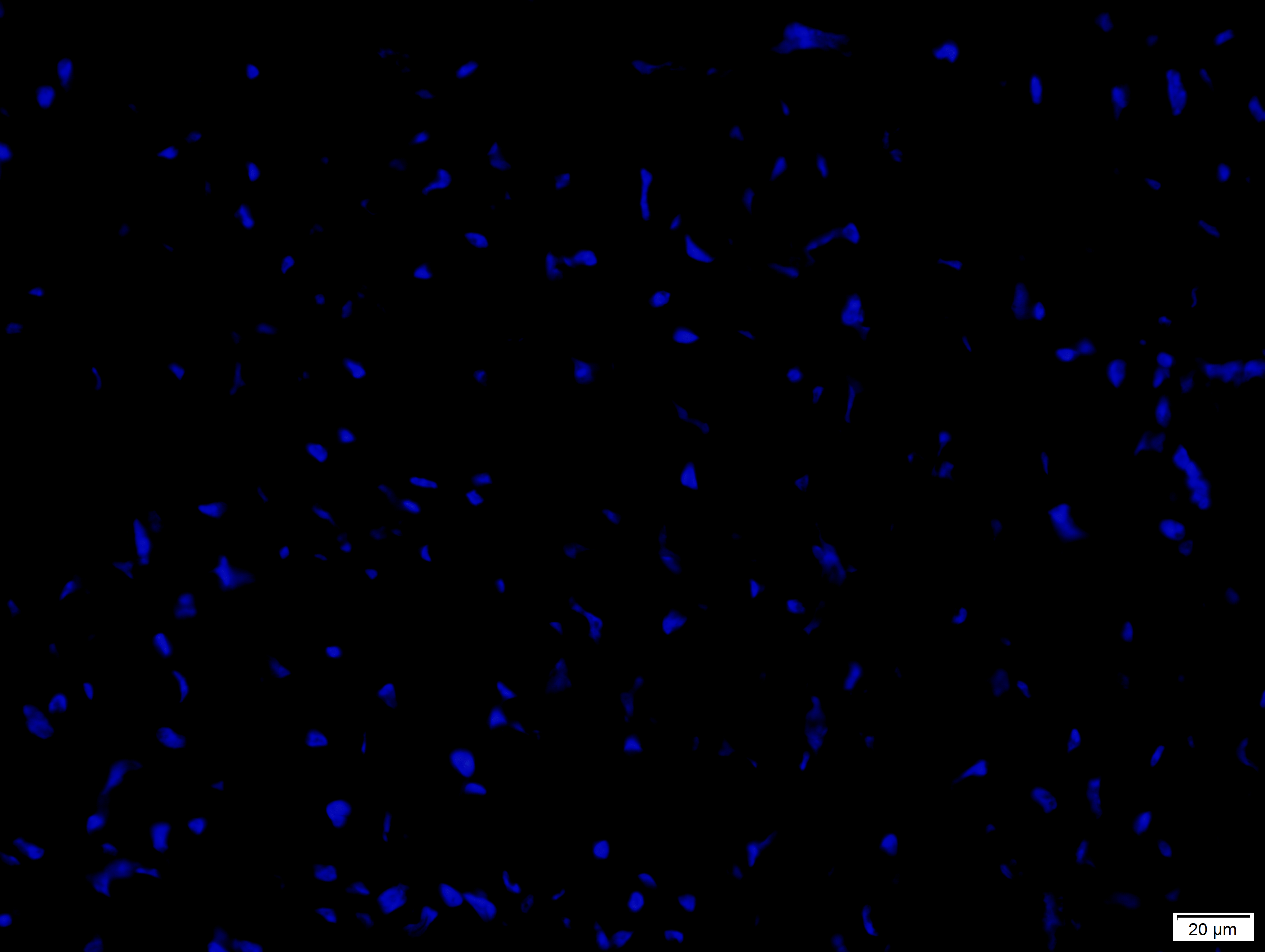

Supplement: Supplementary file 1 [file DataSheet3.ZIP › Supplement data 3/FIGURE 4/FIGURE 4A-B/microscopy images/ex527-400x-DAPI-2ms.tif]

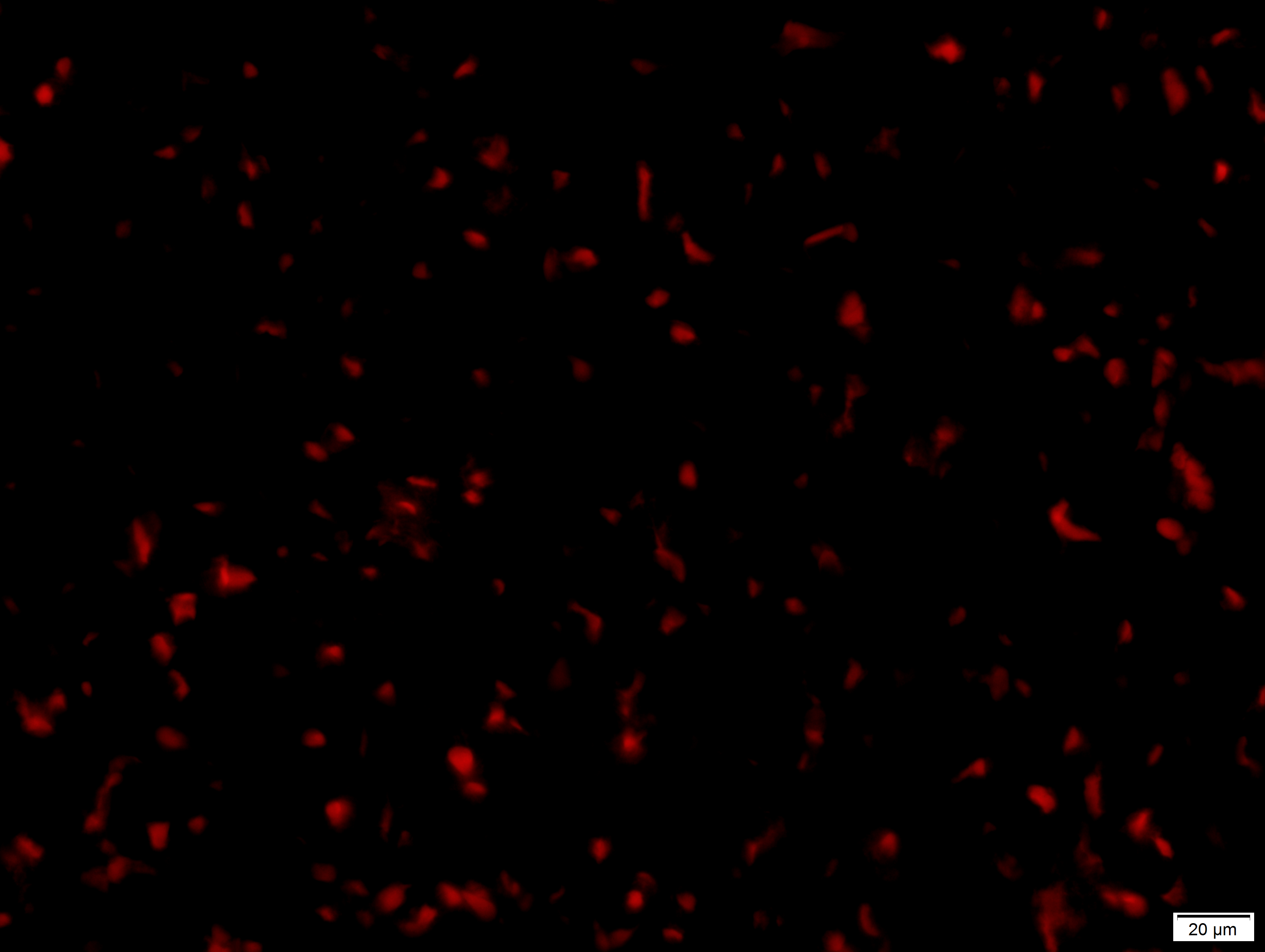

Supplement: Supplementary file 1 [file DataSheet3.ZIP › Supplement data 3/FIGURE 4/FIGURE 4A-B/microscopy images/ex527-400x-DHE-50ms.tif]

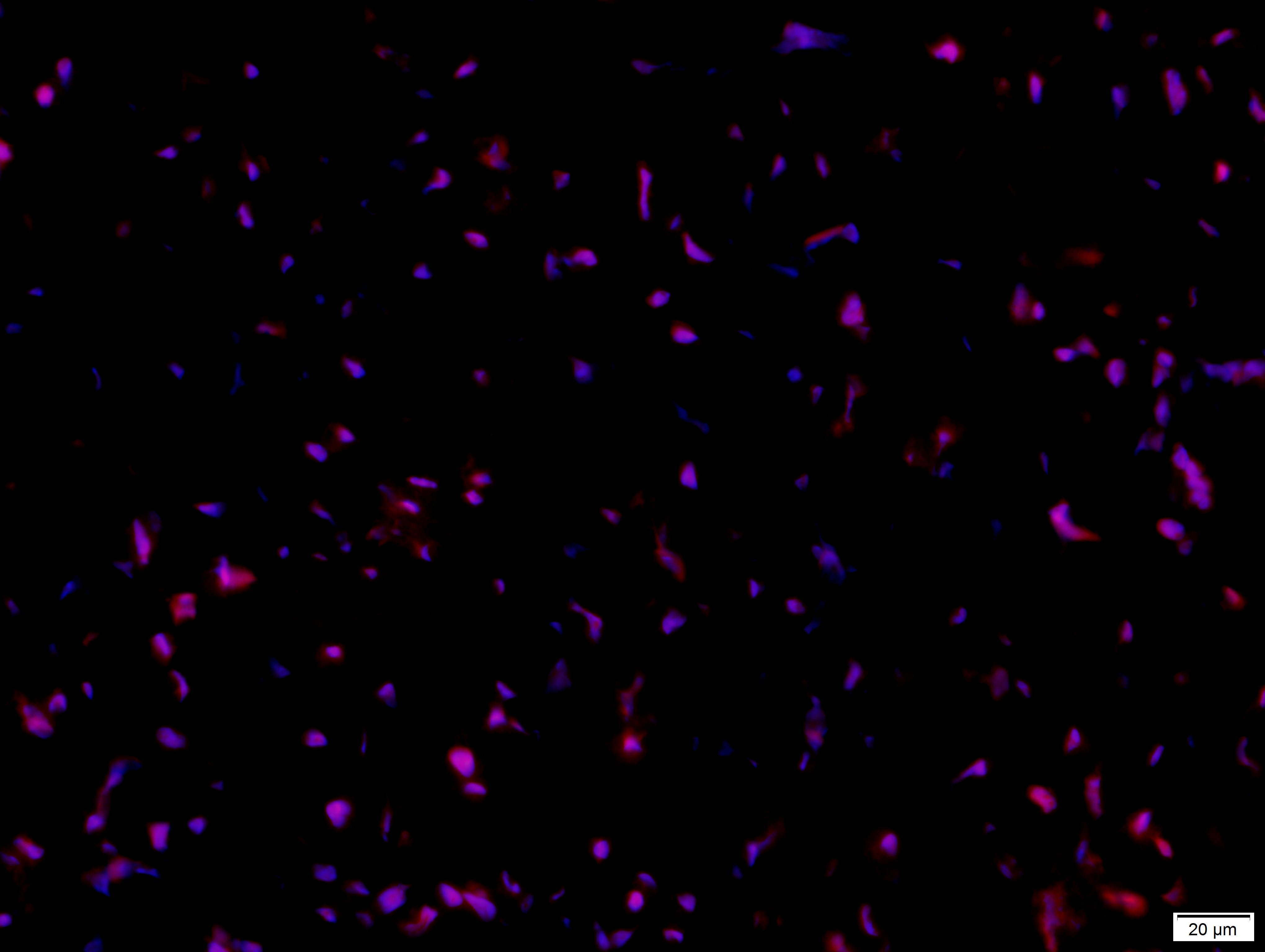

Supplement: Supplementary file 1 [file DataSheet3.ZIP › Supplement data 3/FIGURE 4/FIGURE 4A-B/microscopy images/ex527-400x-merge.tif]

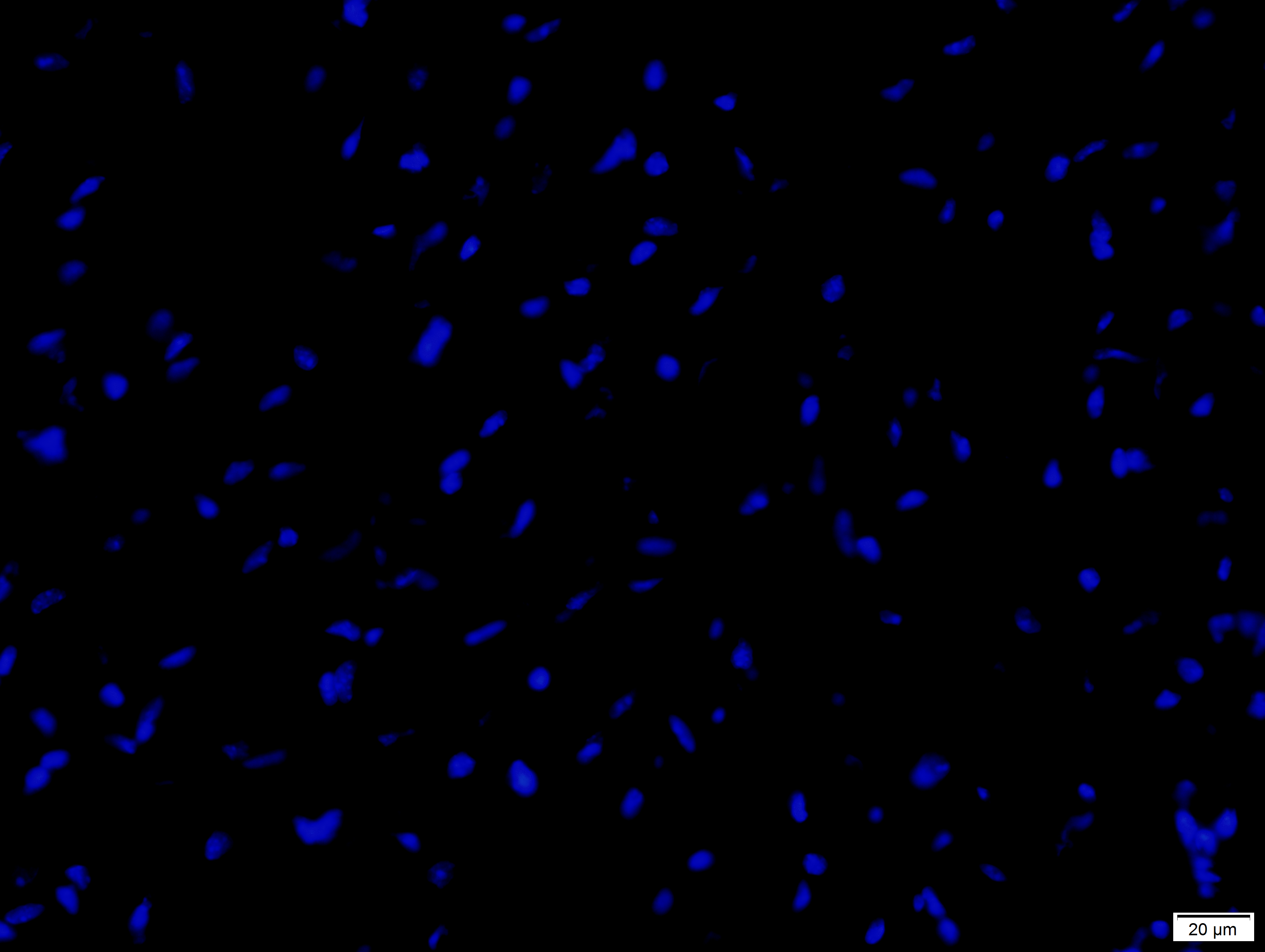

Supplement: Supplementary file 1 [file DataSheet3.ZIP › Supplement data 3/FIGURE 4/FIGURE 4A-B/microscopy images/sham-400x-DAPI-2ms.tif]

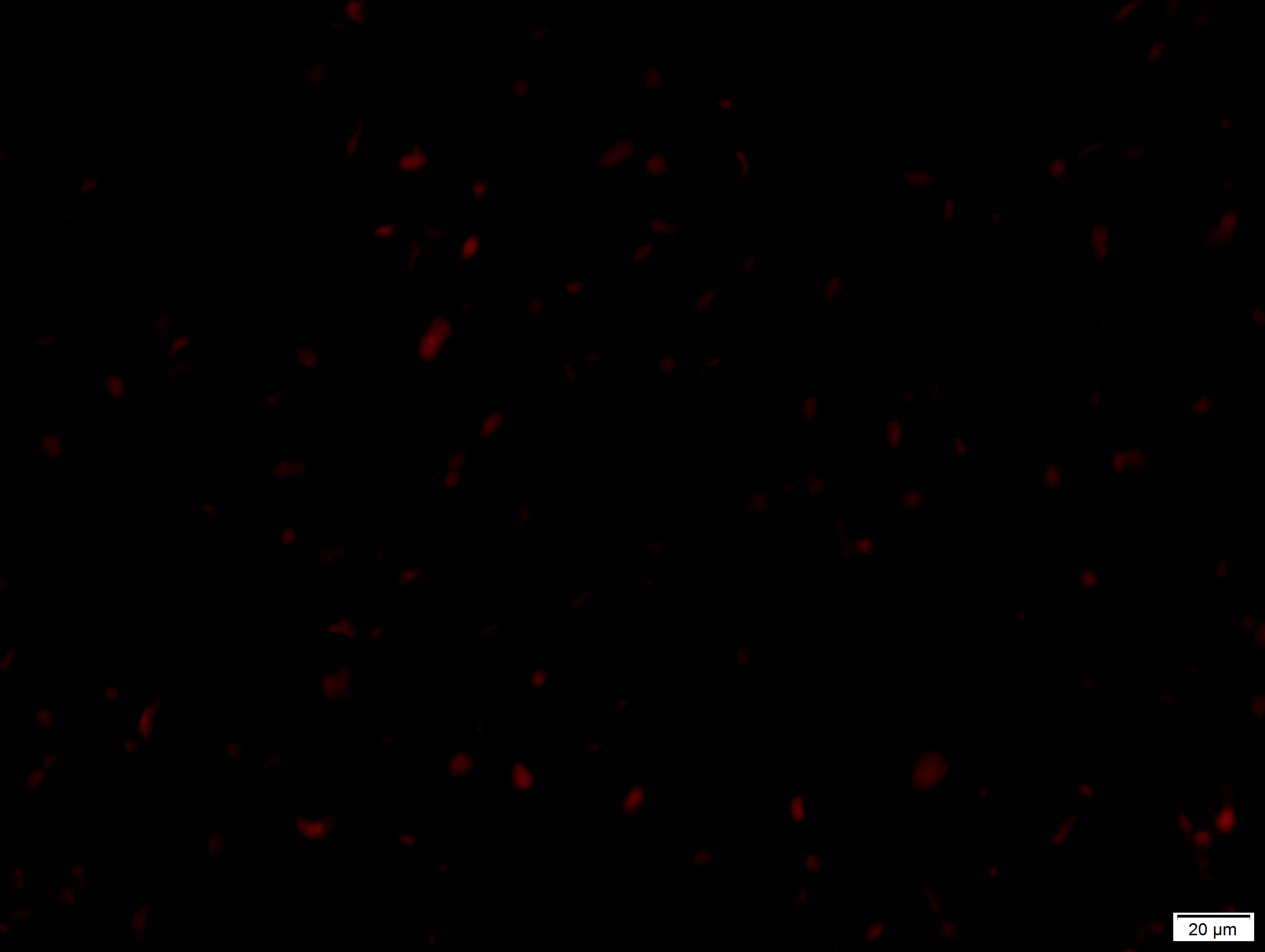

Supplement: Supplementary file 1 [file DataSheet3.ZIP › Supplement data 3/FIGURE 4/FIGURE 4A-B/microscopy images/sham-400x-DHE-50ms.tif]

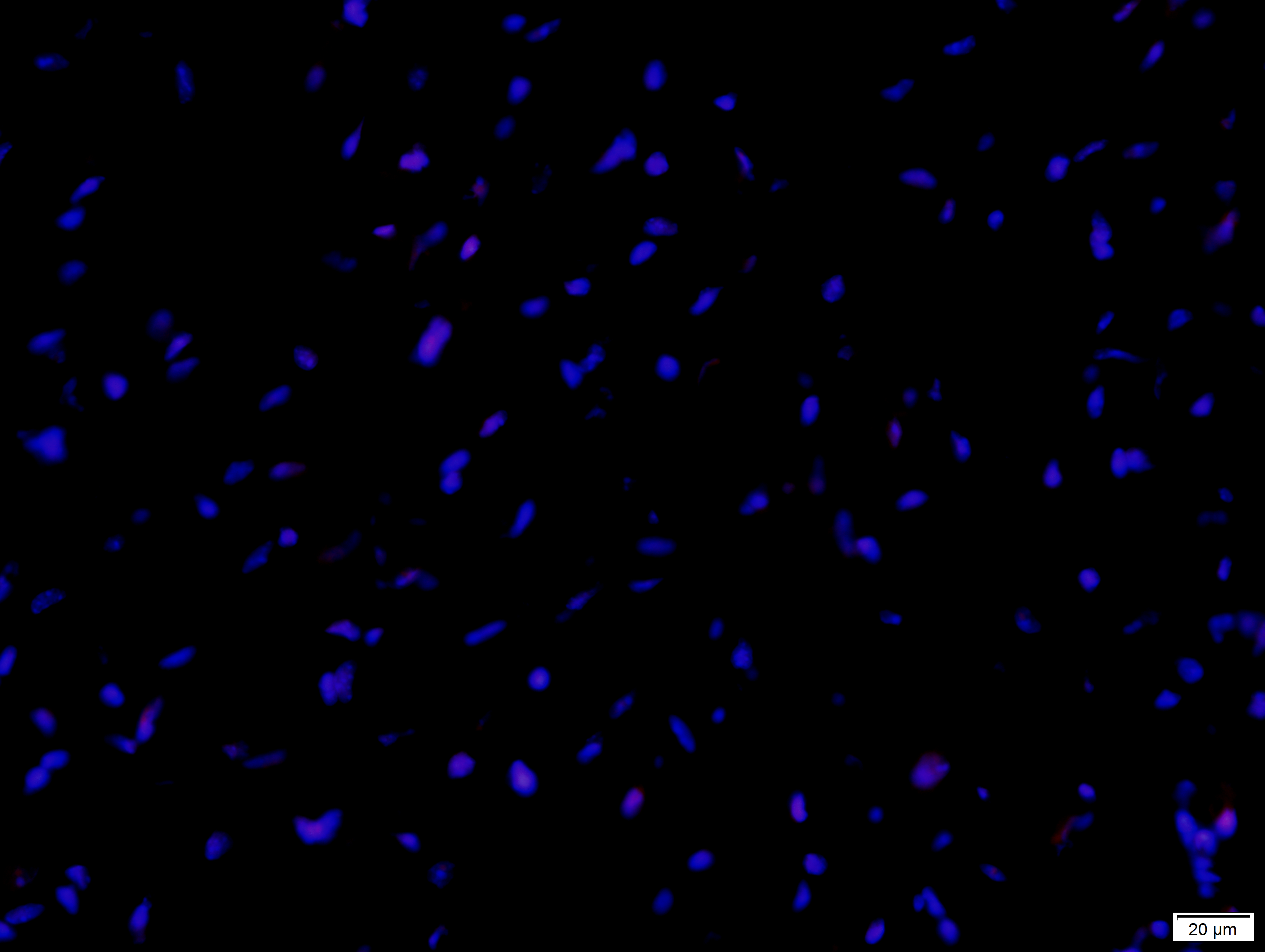

Supplement: Supplementary file 1 [file DataSheet3.ZIP › Supplement data 3/FIGURE 4/FIGURE 4A-B/microscopy images/sham-400x-merge.tif]

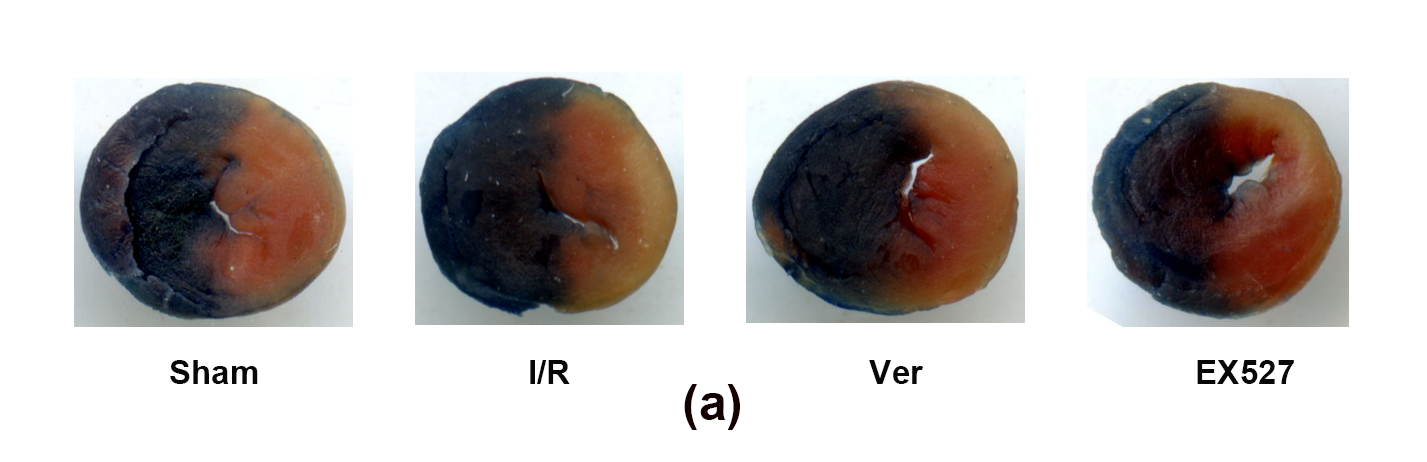

Supplement: Supplementary file 2 [file DataSheet4.ZIP › Supplement data 4/FIGURE 6/FIGURE 6A.tif]

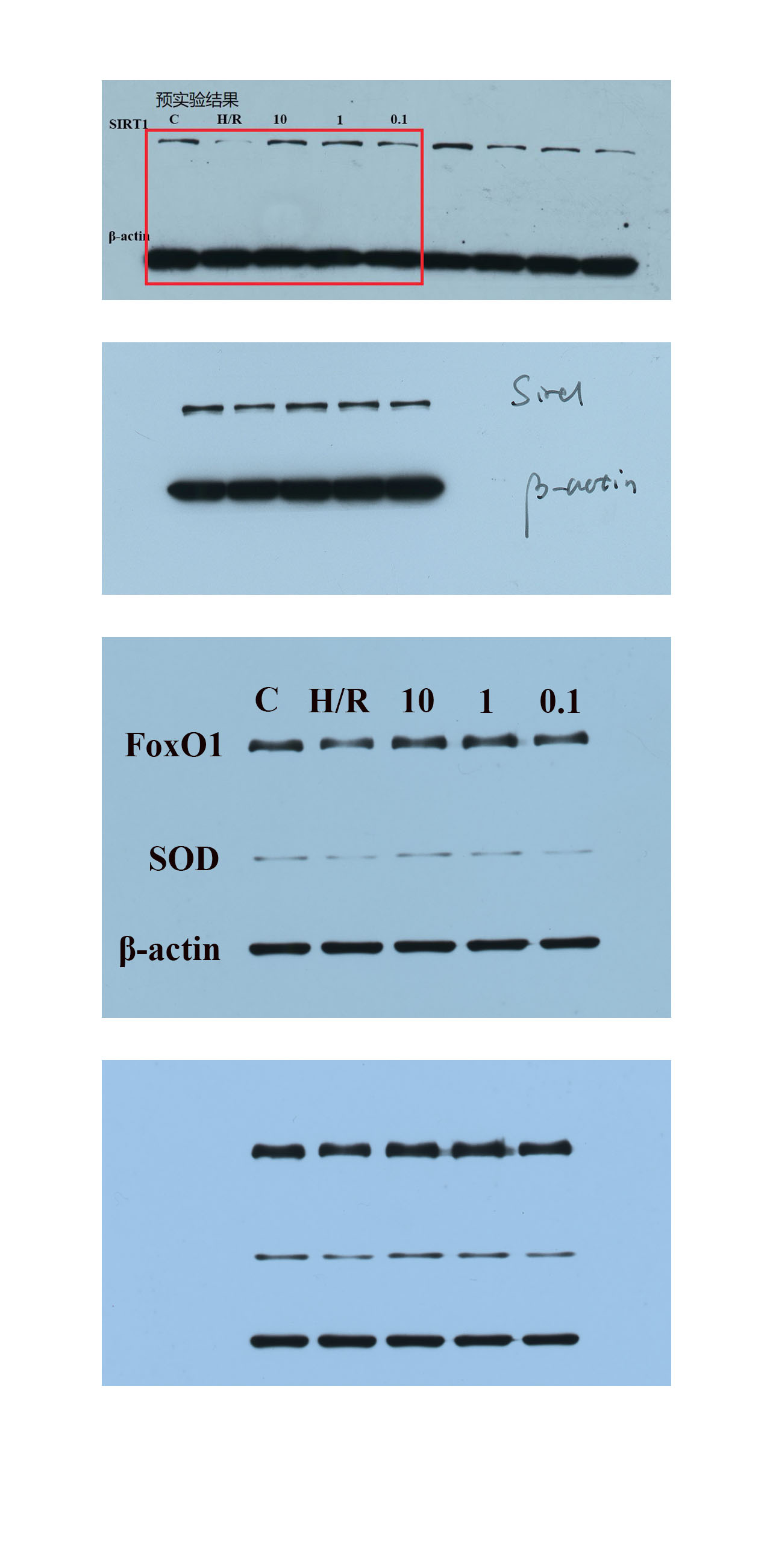

Supplement: Supplementary file 3 [file DataSheet1.ZIP › Supplement data 1/FIGURE 1/FIGURE 1A-D/FIGURE1-JPG.jpg]

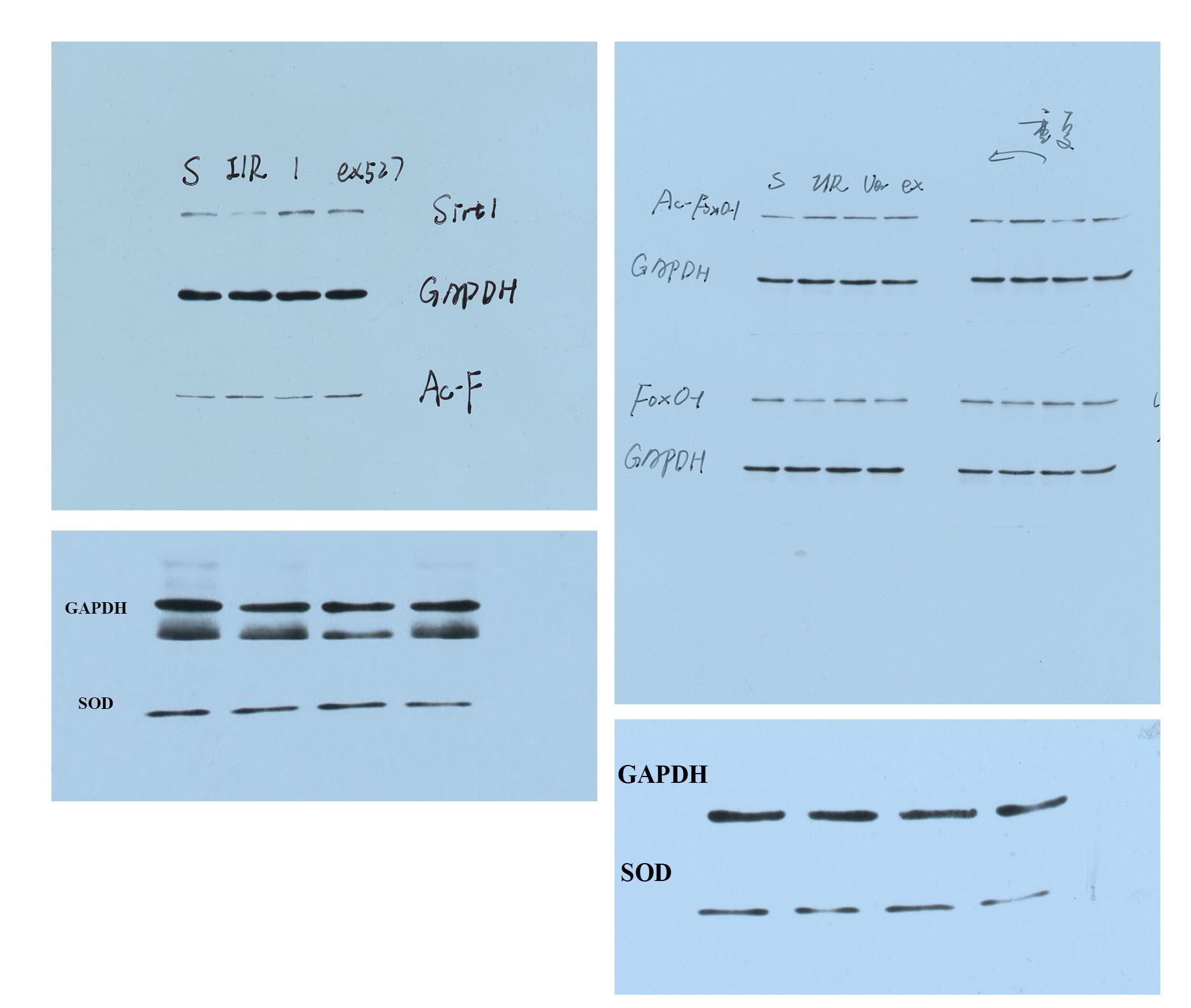

Supplement: Supplementary file 3 [file DataSheet1.ZIP › Supplement data 1/FIGURE 3/FIGURE 3/FIGURE3-JPG.jpg]

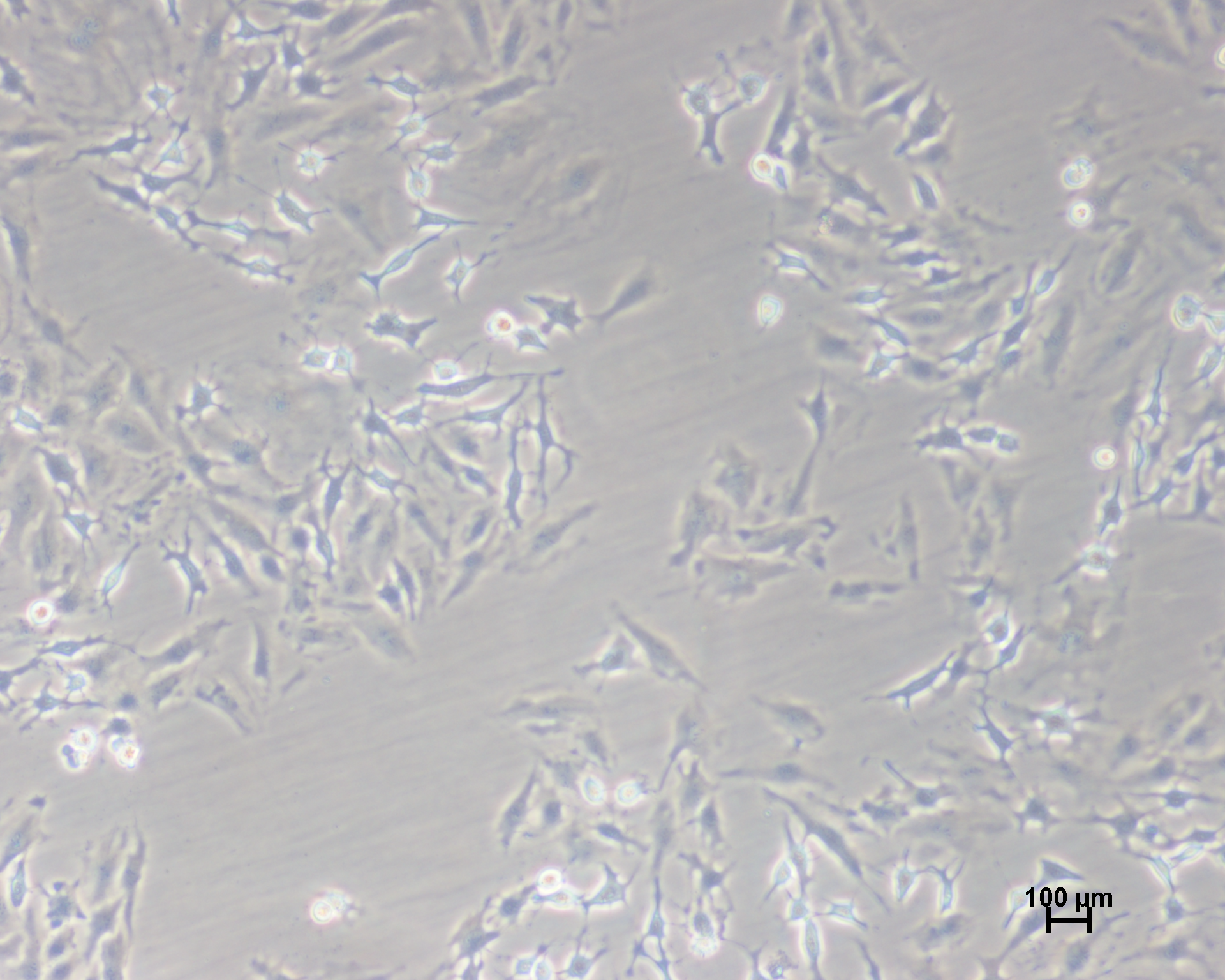

Supplement: Supplementary file 4 [file DataSheet2.ZIP › Supplement data 2/FIGURE 2/FIGURE 2A/HR+VER+EX527.tif]

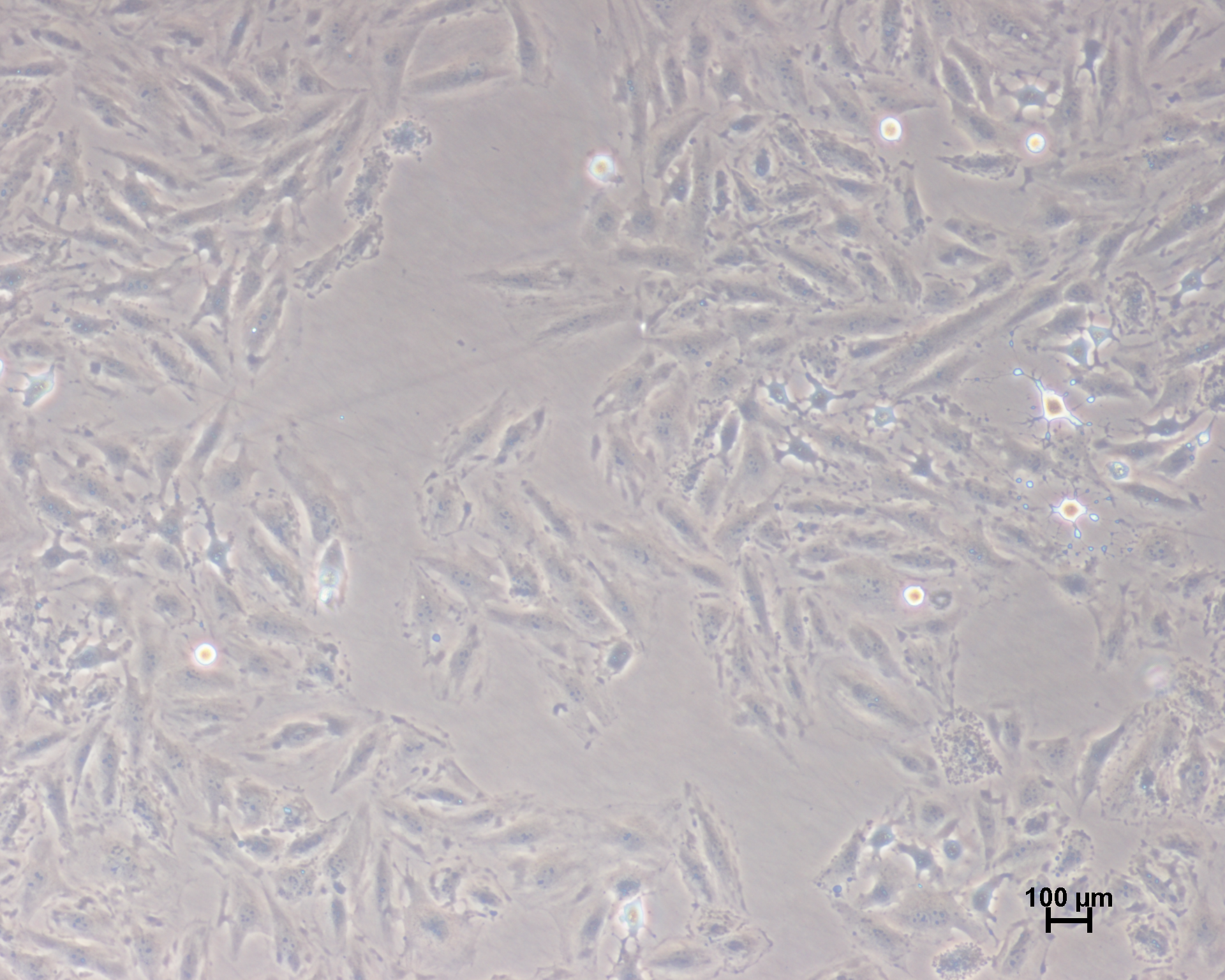

Supplement: Supplementary file 4 [file DataSheet2.ZIP › Supplement data 2/FIGURE 2/FIGURE 2A/HR+VER.tif]

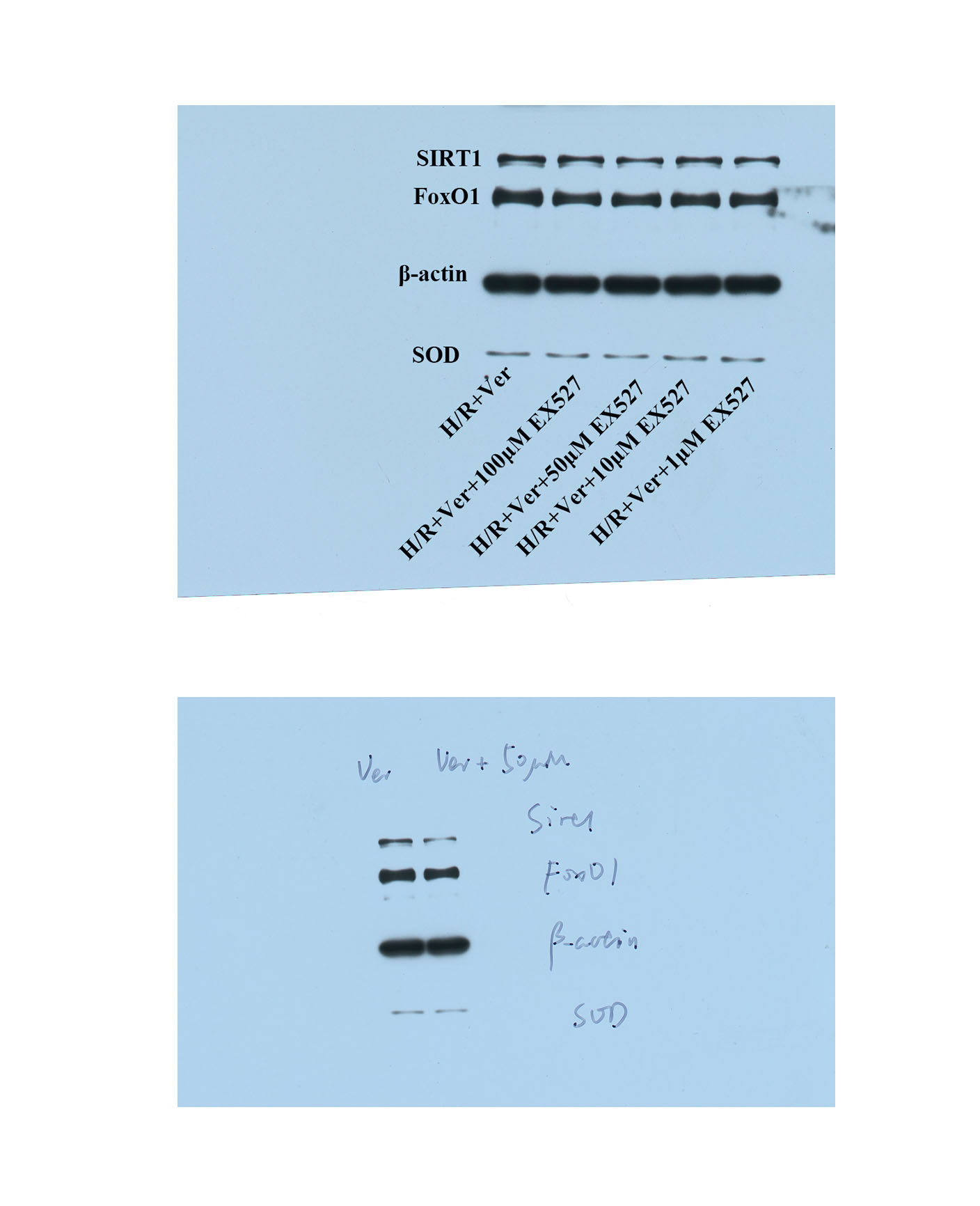

Supplement: Supplementary file 4 [file DataSheet2.ZIP › Supplement data 2/FIGURE 2/FIGURE 2B-E/FIGURE2-JPG.jpg]

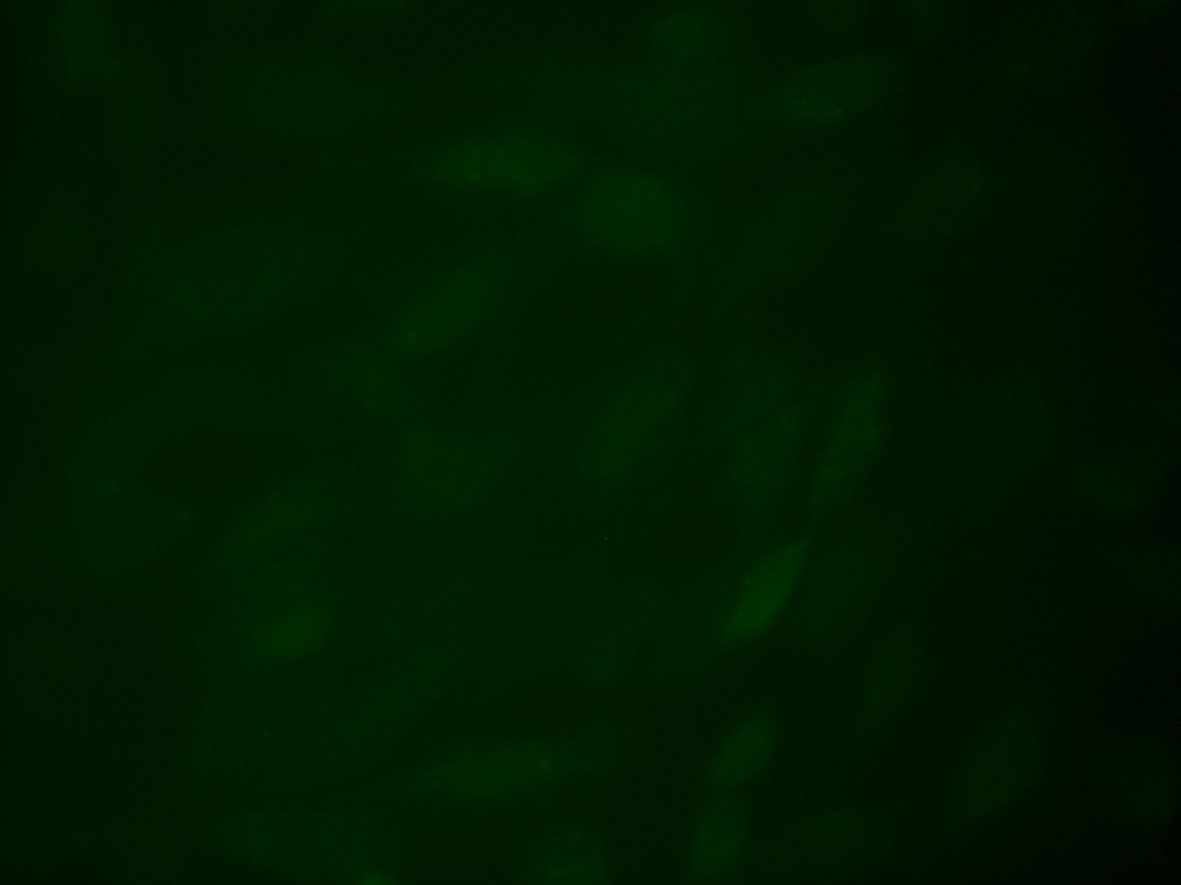

Supplement: Supplementary file 5 [file DataSheet5.ZIP › Supplementdata5/Control/Con1.jpg]

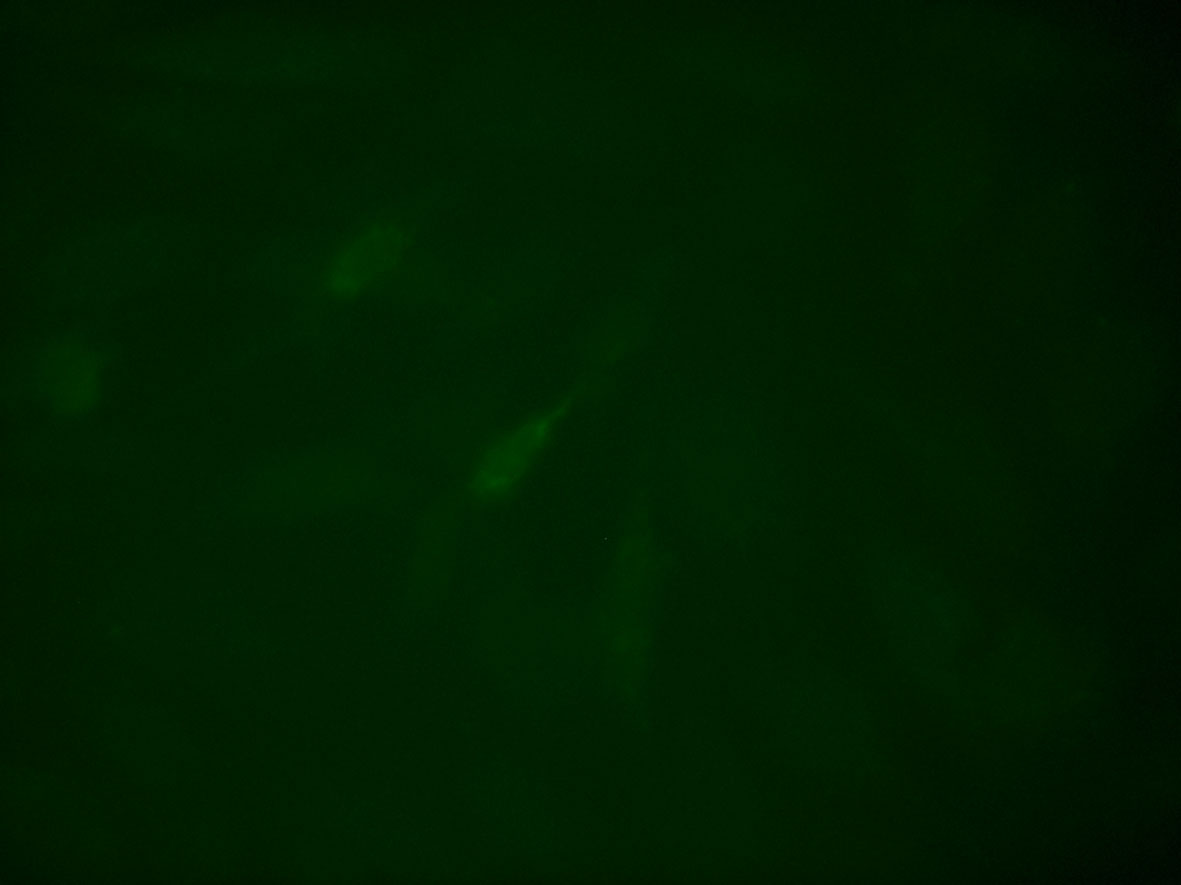

Supplement: Supplementary file 5 [file DataSheet5.ZIP › Supplementdata5/Control/Con2.jpg]

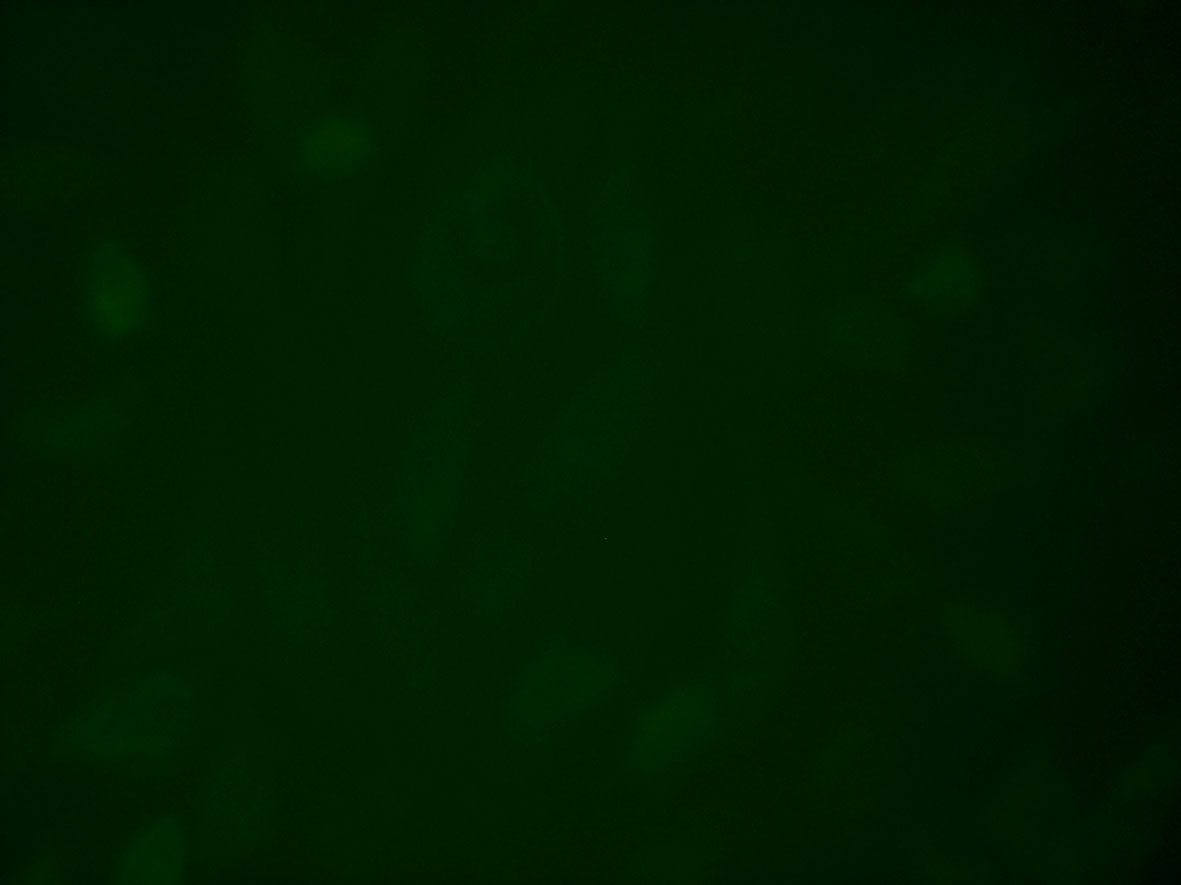

Supplement: Supplementary file 5 [file DataSheet5.ZIP › Supplementdata5/Control/Con3.jpg]

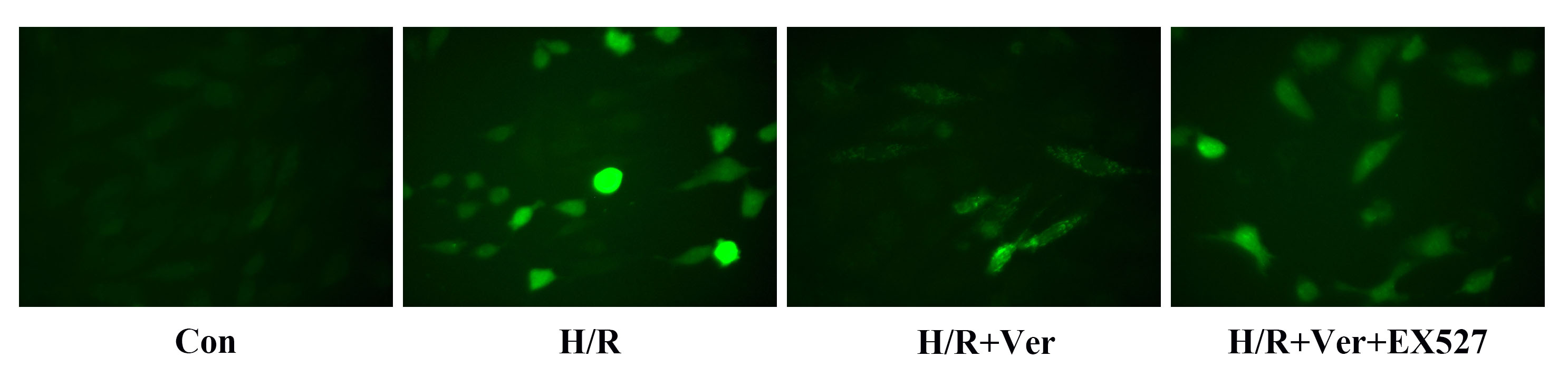

Supplement: Supplementary file 5 [file DataSheet5.ZIP › Supplementdata5/DCFH-DA.jpg]

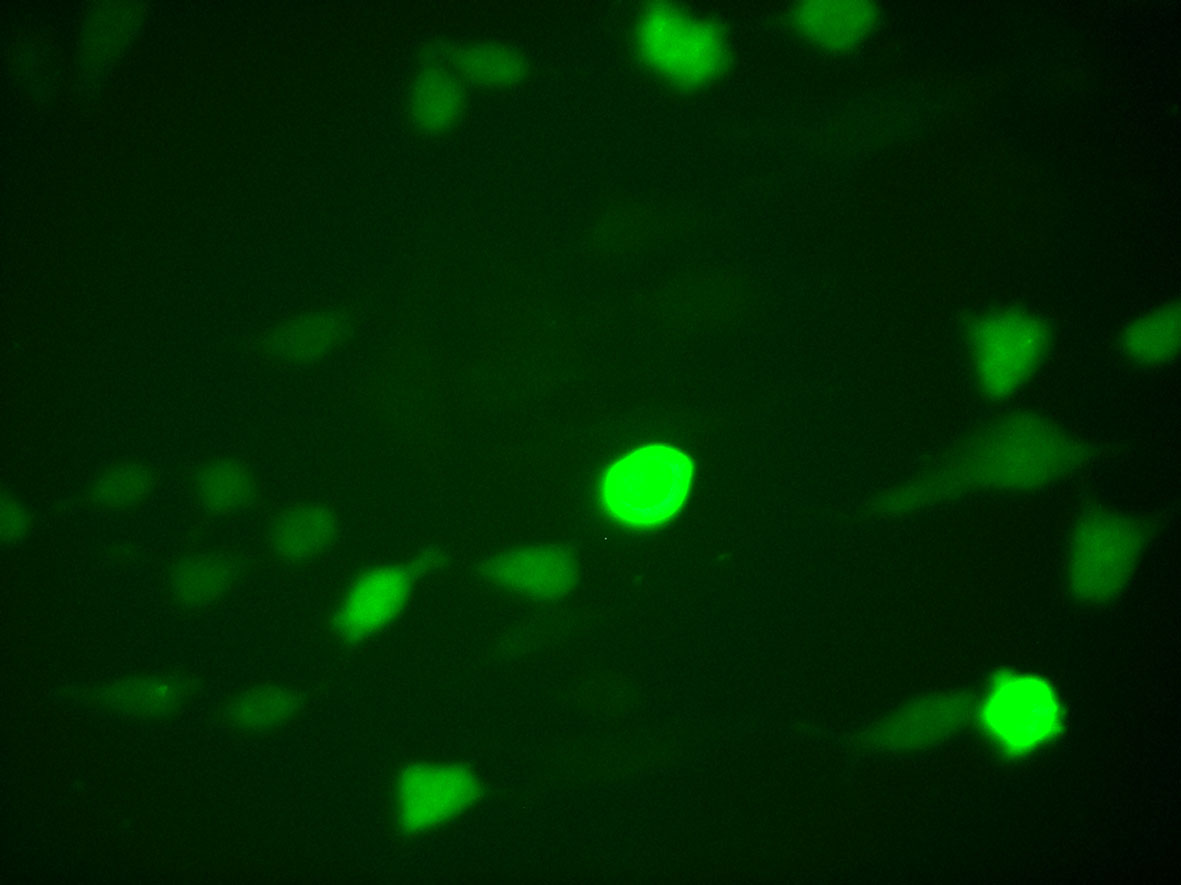

Supplement: Supplementary file 5 [file DataSheet5.ZIP › Supplementdata5/HR/HR1.jpg]

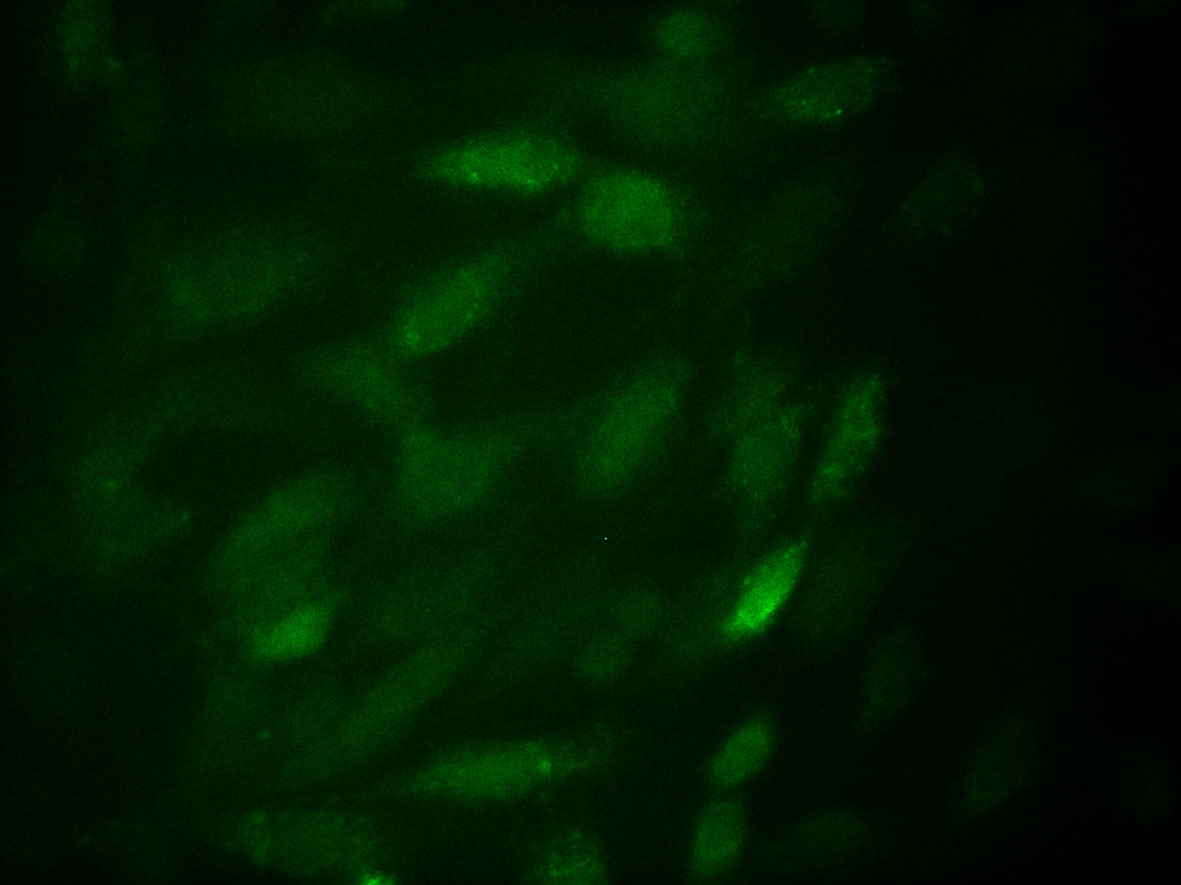

Supplement: Supplementary file 5 [file DataSheet5.ZIP › Supplementdata5/HR/HR2.jpg]

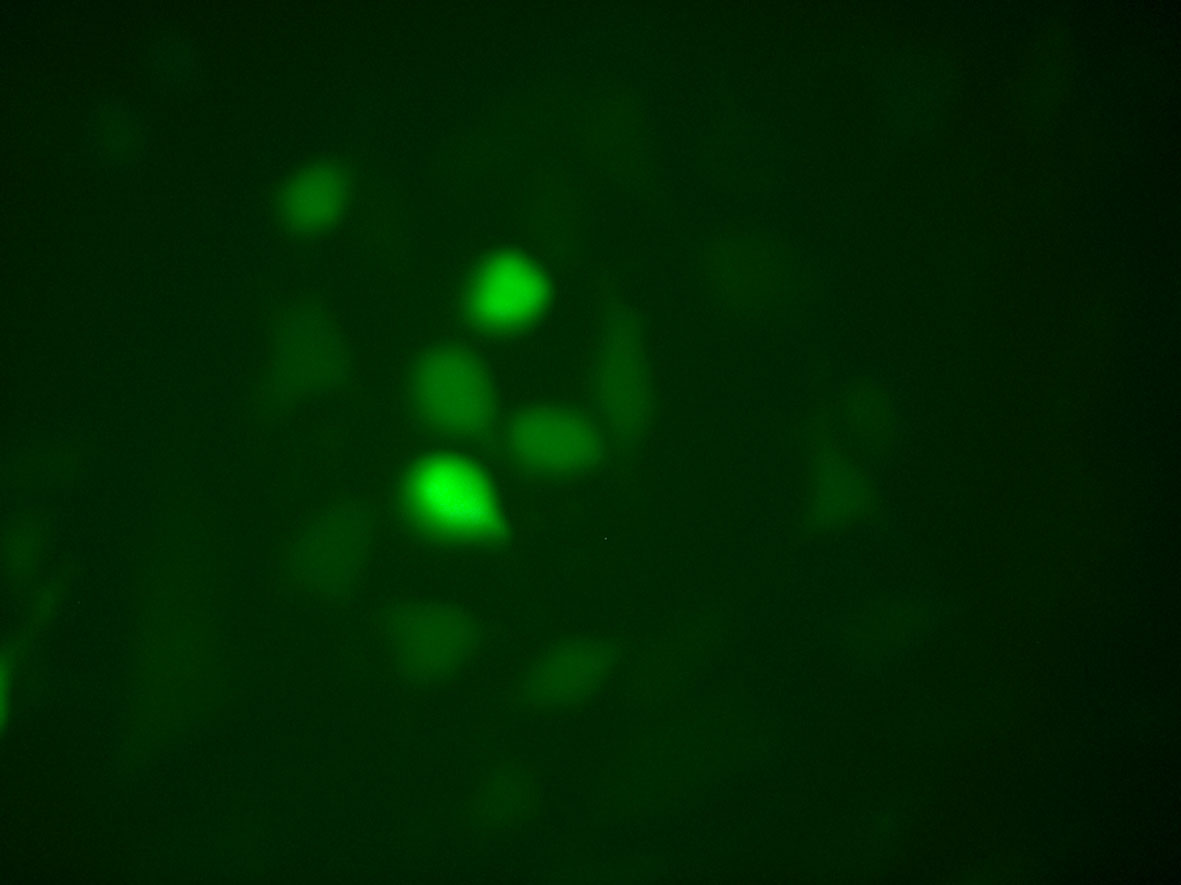

Supplement: Supplementary file 5 [file DataSheet5.ZIP › Supplementdata5/HR/HR3.jpg]

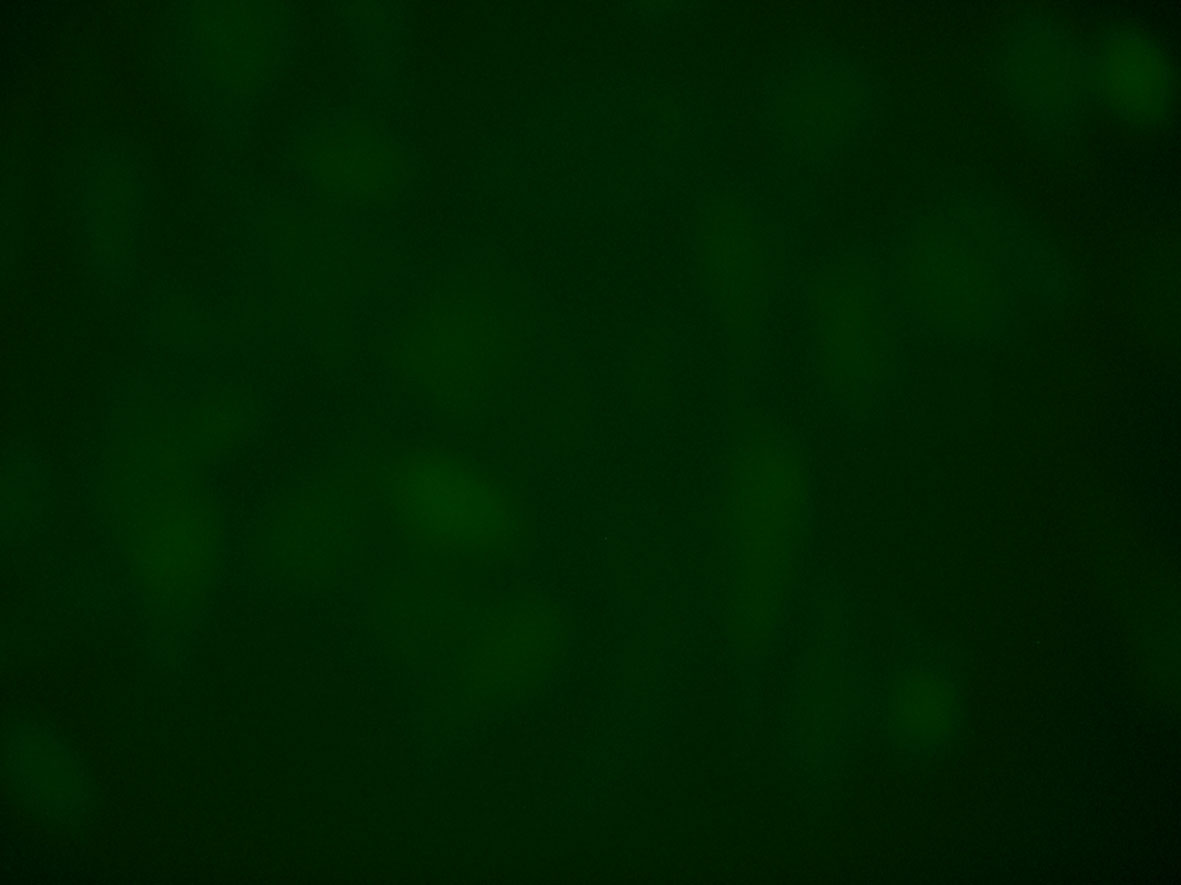

Supplement: Supplementary file 5 [file DataSheet5.ZIP › Supplementdata5/HR+Ver/HR+Ver1.jpg]

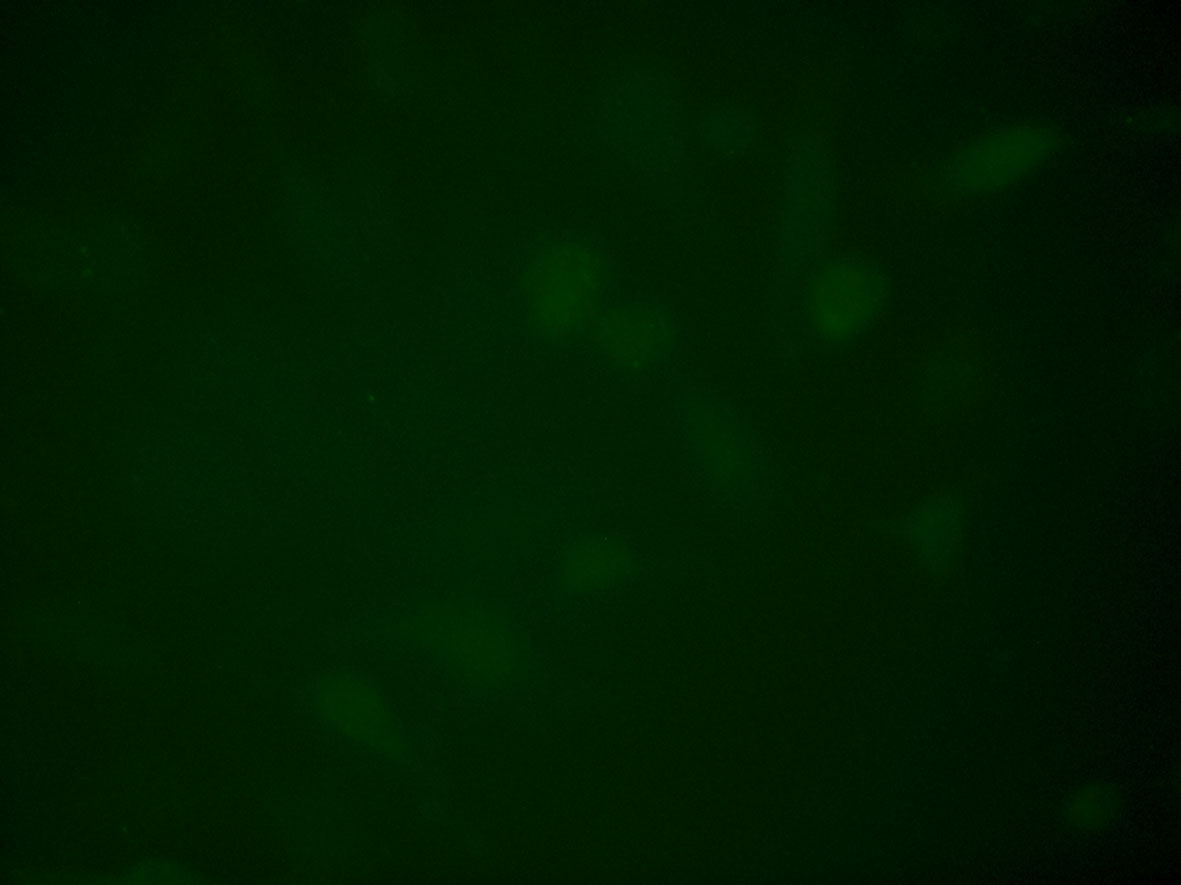

Supplement: Supplementary file 5 [file DataSheet5.ZIP › Supplementdata5/HR+Ver/HR+Ver2.jpg]

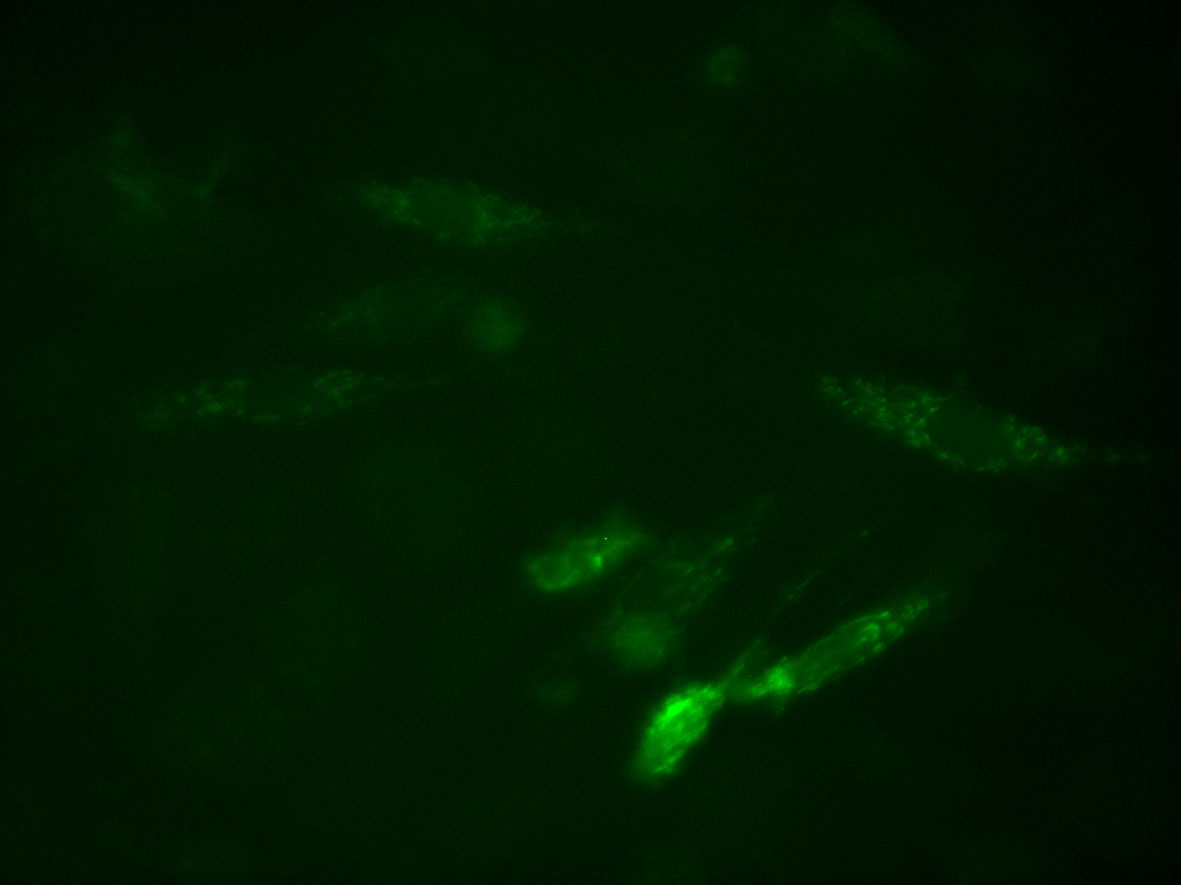

Supplement: Supplementary file 5 [file DataSheet5.ZIP › Supplementdata5/HR+Ver/HR+Ver3.jpg]

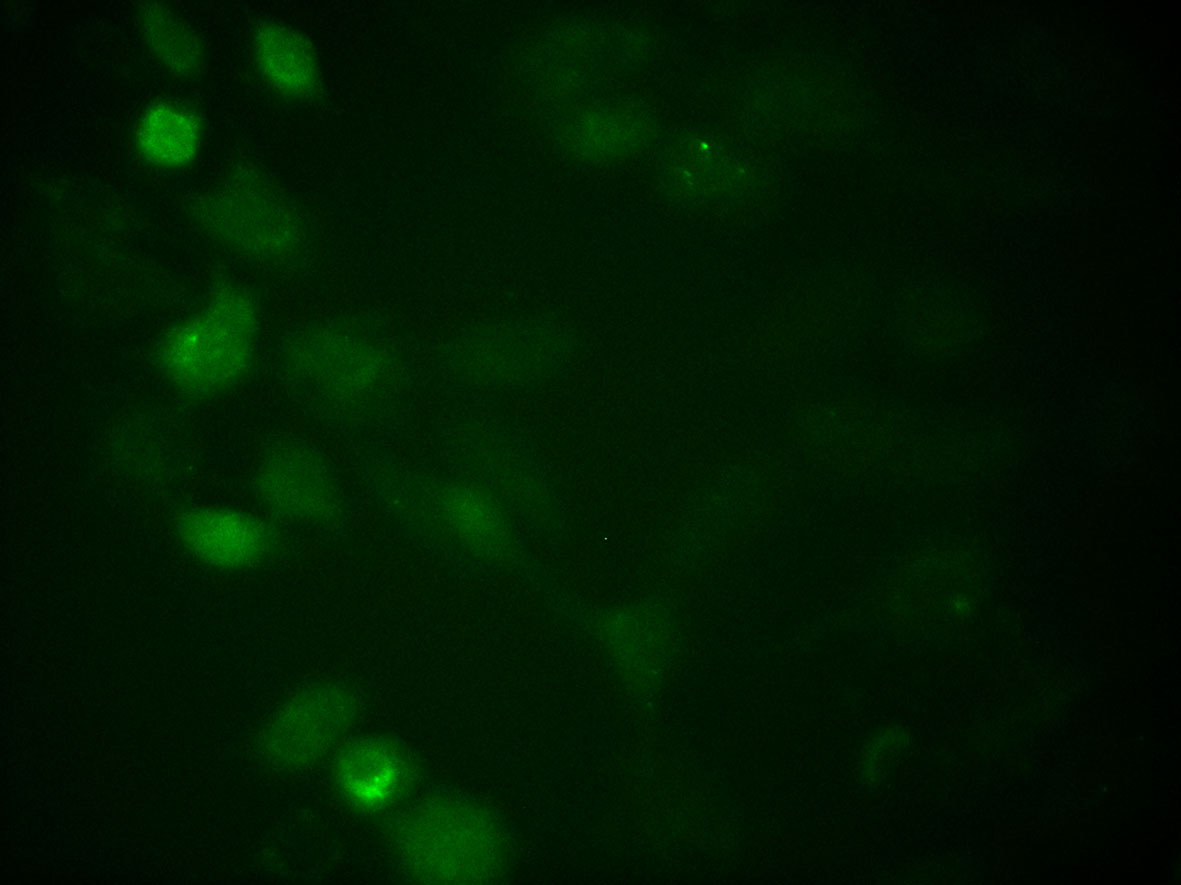

Supplement: Supplementary file 5 [file DataSheet5.ZIP › Supplementdata5/HR+Ver+EX527/HR+Ver+EX527-1.jpg]

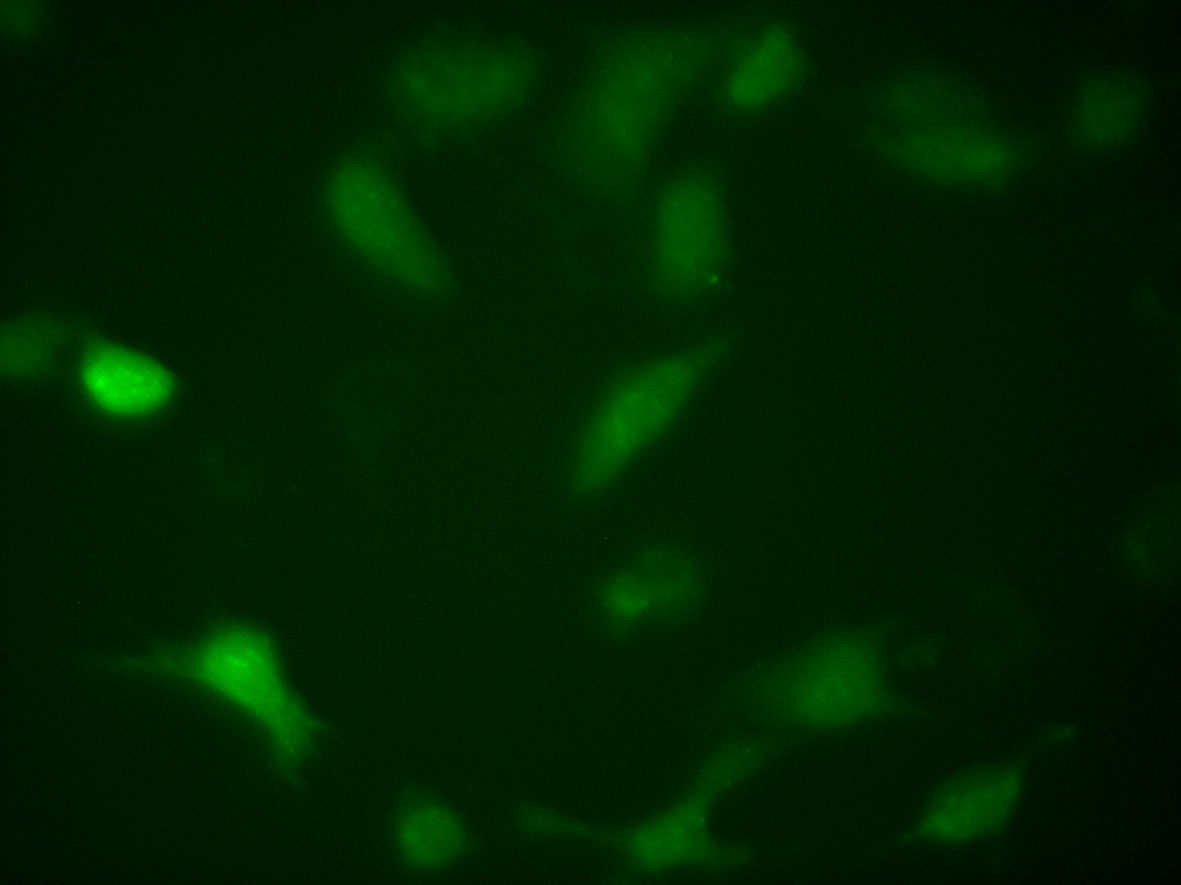

Supplement: Supplementary file 5 [file DataSheet5.ZIP › Supplementdata5/HR+Ver+EX527/HR+Ver+EX527-2.jpg]

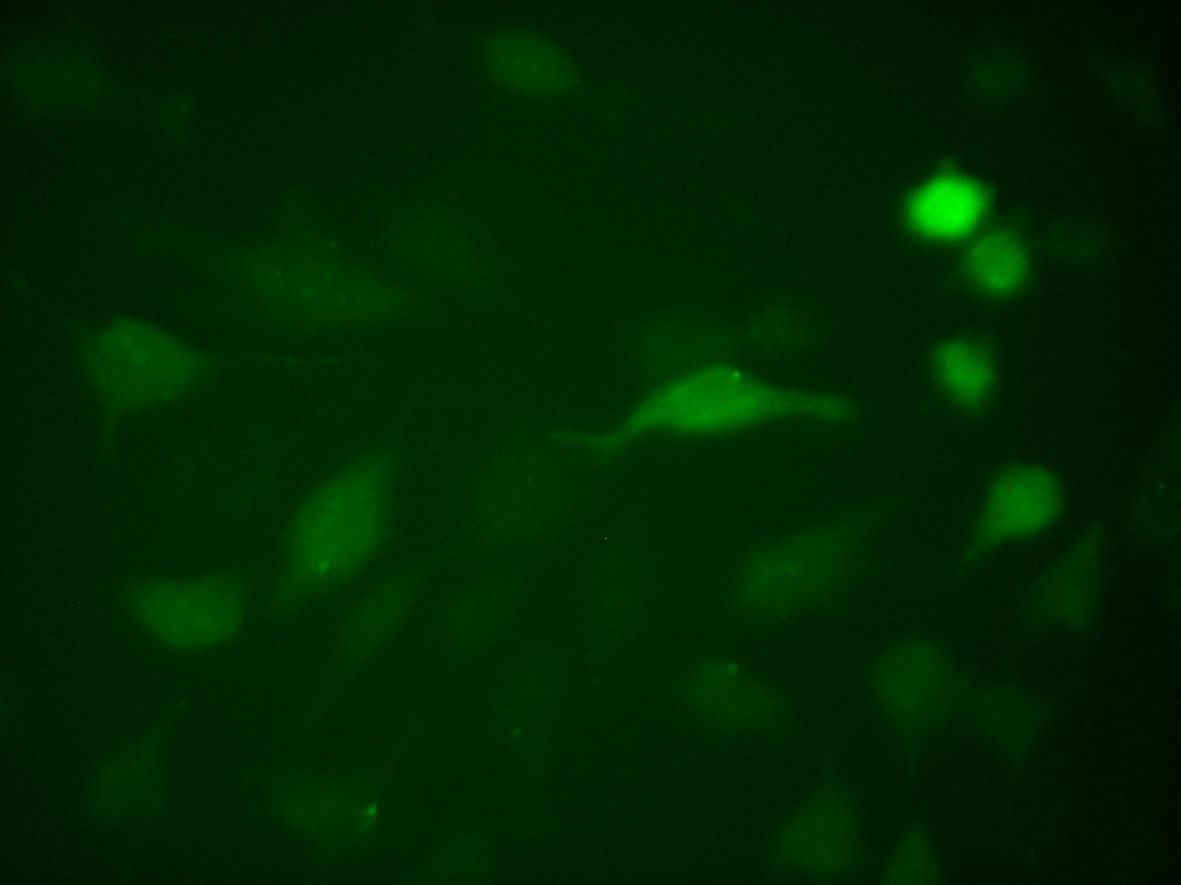

Supplement: Supplementary file 5 [file DataSheet5.ZIP › Supplementdata5/HR+Ver+EX527/HR+Ver+EX527-3.jpg]
